# Sting and p53 DNA repair pathways are compromised in Alzheimer’s disease

**DOI:** 10.1038/s41598-023-35533-6

**Published:** 2023-05-23

**Authors:** Thomas J. Nelson, Yunhui Xu

**Affiliations:** Department of Neurology, Marshall University Joan C. Edwards School of Medicine, Huntington, WV 25704 USA

**Keywords:** Biochemistry, Neuroscience, Neurology, Pathogenesis

## Abstract

Alzheimer’s disease (AD) is the most common cause of dementia. A common finding in AD is DNA damage. Double-strand DNA breaks (DSBs) are particularly hazardous to neurons because their post-mitotic state forces neurons to rely on error-prone and potentially mutagenic mechanisms to repair DNA breaks. However, it remains unclear whether DNA damage results from increased DNA damage or failure of DNA repair. Oligomerization of the tumor suppressor protein p53 is an essential part of DSB repair, and p53 phosphorylated on S15 is an indicator of DNA damage. We report that the monomer:dimer ratio of phosphorylated (S15) p53 is increased by 2.86-fold in temporal lobes of AD patients compared to age-matched controls, indicating that p53 oligomerization is compromised in AD. In vitro oxidation of p53 with 100 nM H_2_O_2_ produced a similar shift in the monomer:dimer ratio. A COMET test showed a higher level of DNA degradation in AD consistent with double-strand DNA damage or inhibition of repair. Protein carbonylation was also elevated (190% of control), indicating elevated oxidative stress in AD patients. Levels of the DNA repair support protein 14-3-3σ, γ-H2AX, a phosphorylated histone marking double strand DNA breaks, and phosphorylated ataxia telangiectasia mutated (ATM) protein were all increased. cGAS-STING-interferon signaling was impaired in AD and was accompanied by a depletion of STING protein from Golgi and a failure to elevate interferon despite the presence of DSBs. The results suggest that oxidation of p53 by ROS could inhibit the DDR and decrease its ability to orchestrate DSB repair by altering the oligomerization state of p53. The failure of immune-stimulated DNA repair may contribute to cell loss in AD and suggests new therapeutic targets for AD.

## Introduction

The four most important predictive factors for AD are obesity, cardiovascular disease, age, and ApoE4 genotype^1^. What these four factors have in common is the ability to induce or promote DNA damage, which results in chronic inflammation, cell loss, and cellular senescence. DNA damage has long been known to be prominent in AD^2–10^ and can lead to failed cell cycle re-entry, cellular senescence, or p53-mediated apoptosis^11^. Widespread somatic gene recombination, possibly initiated by double strand breaks (DSBs), has also been observed in AD, including intra-exonic junctions, insertions, and deletions^9^. There are also reports that ApoE4 interacts in a manner not yet fully understood through thioredoxin^12^ and redox-factor 1 to inhibit activation of tumor suppressor protein p53. Upon activation, p53 tetramerizes and binds to DNA, where it participates in DNA repair. Phosphorylation at S15 occurs after DNA damage and leads to decreased interaction with its negative regulator, MDM2^13^, which targets p53 for ubiquitination and proteasomal degradation.

Oxidative DNA damage accumulates with age^14^, and DSBs are a potential driver for aging^15–17^. DSBs are particularly hazardous to neurons because their post-mitotic state forces neurons to rely on the error-prone and potentially mutagenic mechanism of non-homologous end-joining (NHEJ) instead of error-free homologous recombination (HR) to repair DNA breaks^18,19^. Oxidative stress, which is an important cause of DNA damage, is a key mediator of neuroinflammation^20–22^ and cardiovascular disease^23^. Viruses also induce ROS and cytokine release by acting on pattern recognition receptors (PRRs). A high-fat diet creates DSBs via inflammatory cytokines which interfere with DSB rejoining^24,25^. Thus, DNA damage can occur from several seemingly unrelated pathways that are implicated as risk factors for AD.

There is strong evidence that p53 is involved AD pathogenesis^26–31^. p53 is a key component of DSB repair. It has strong effects on both the innate and adaptive immune systems^32^ and participates in the inflammatory response to DSBs. In its tetramer form p53 binds DNA, while monomers and loss-of-function mutations, as occur in cancer, contribute to cell proliferation. Activation of p53 in the CNS can induce tau aggregation and neurofibrillary tangles^33^ and loss of dendritic spines^34^. Conformationally altered p53 in lymphocytes, aka unfolded/aggregated p53, has been proposed as a peripheral marker of early AD^35–43^; however, unfolded p53 has not yet been found in brain. p53 is also activated in obesity^44,45^ due to oxidative DNA damage^46–48^.

Foreign or fragmented double-strand DNA (dsDNA) in the cytosol caused by viral infections or DNA damage is recognized by the DNA sensor DNA-PKcs (DNA-dependent protein kinase catalytic subunit), which phosphorylates and activates the enzyme cGAS (cyclic GMP-AMP [cGAMP] synthase)^49^ to produce the cyclic dinucleotide 2′,3′-cGAMP. cGAMP induces dimerization and activation of STING (Stimulator of Interferon Gene), which translocates from the endoplasmic reticulum to the Golgi and binds to the kinases TBK1 (TANK-binding kinase 1 and IKKβ (IkappaB kinase-β). These kinases activate IRF3 (interferon regulatory factor 3) and NF-κB (nuclear factor kappa-light-chain-enhancer of activated B cells) to induce the expression of interferon-stimulated genes. STING can also stimulate autophagy, necroptosis, and possibly ferroptosis^50^ and can induce cellular senescence^51–53^, all of which are implicated in AD^54^.

In this study we found that p53 oligomerization is inhibited in AD. Monomerization is induced by oxidants such as H_2_O_2_. The cGAS-STING interferon response in AD is also inhibited due to depletion of STING from Golgi despite high levels of fragmented DNA. The evidence is consistent with impairment of p53 oligomerization from chronic oxidative DNA damage and compromise of the Type I interferon response in AD by interference with TBK1 phosphorylation of IRF3, as occurs in cancer cells.

## Materials and methods

### Human autopsy samples

Frozen anonymized samples of temporal lobe (Brodmann’s Area 38) obtained from AD and unaffected patients at autopsy were acquired from NeuroBioBank through the Human Brain and Spinal Fluid Resource Center, VA W Los Angeles Healthcare Center, Los Angeles, CA. Average age was not significantly different (AD = 77.2 ± 1.85, Control = 76.3 ± 1.9, ${\overline{\text{x}}}$ ± SEM, p = 0.74). Postmortem intervals were also not significantly different between AD and unaffected patients (AD = 14.33 ± 0.73 h, Control = 15.05 ± 0.98 h, n = 20, p = 0.56; n = 20, ${\overline{\text{x}}}$ ± SEM). Both AD and unaffected aged patients had a comparable incidence of hypertension (6/20, 30%), and 50% of patients diagnosed with AD (10/20) also had atherosclerosis or cardiovascular disease compared with 35% (7/20) of controls (*Χ*^2^(1, n = 20) = 0.41, p = 0.52). All experiments were performed in accordance with all relevant guidelines and regulations. In previous experiments, 40% of AD patients but no controls had at least one allele of ApoE4, a known risk factor for AD, obesity, and metabolic syndrome^55^ (p = 0.003).

### Comet assay

Nuclei from human autopsy temporal lobe samples from AD and age-matched controls were isolated by standard methods. Briefly, 10–20 mg tissue was gently homogenized at 4 °C in 9 volumes of NILR (Nuclear Isolation Lysis Reagent = 10 mM Tris–HCl pH 7.5, 10 mM NaCl, 3 mM MgCl_2_, 0.1% NP40, 0.25 M sucrose) in the presence of 1 × HALT protease/phosphatase inhibitors (ThermoFisher 78443) in a motorized Teflon homogenizer at 200 rpm. The homogenate was centrifuged at 4 °C and 300*g* for 10 min and the pellet containing enriched nuclei was suspended in 100 µl PBS + 1 × HALT protease/phosphatase inhibitors. Nuclei were counted in a hemacytometer and diluted to 1 × 10^5^ nuclei/ml. Approximately 500–1000 nuclei were added to 45 µl low-melting agarose, plated on Comet slides (Trevigen 4250-050-K), and incubated with lysis solution and alkaline unwinding solution according to the manufacturer’s protocol. DNA was electrophoresed at 21 V and 300 mA for 1 h at 4 °C. After staining with SYBR Gold, slides were illuminated with a 100-mW 402 nm semiconductor laser and imaged at 12 bits/channel with a camera and macro lens behind a 530/43 nm FITC emission filter (Thorlabs) from a fixed distance (40 cm) for 5 s. Images were converted to 16-bit grayscale and tail moment, fraction of DNA in the tail^56^, and tail length were measured in Imal 3.8.8 software. At least 50 nuclei comet tails from each of the ten samples were selected at random for each measurement.

### Dot blots

Tissue samples were homogenized in a Teflon homogenizer at 200 rpm at 1:50 dilution (0.01 g wet weight/0.5 ml) of 10 mM Tris–HCl pH 7.4 containing 1 × HALT protease/phosphatase inhibitors. Homogenates were centrifuged at 15,000*g* in 1.5 ml polypropylene centrifuge tubes for 10 min, transferred to new tubes, and re-centrifuged. Supernatants were injected onto a 4 × 150 mm Inertsil WP300 diol size-exclusion (SEC) HPLC column equilibrated with 0.1 M sodium sulfate + 0.1 M sodium phosphate adjusted to pH 6.65 with phosphoric acid. The flow rate was 0.2 ml/min. The column was calibrated using cytochrome c, hemoglobin, trypsin inhibitor, bovine serum albumin monomer and dimer, ATP, and thyroglobulin. The flow was converted to microdroplets by passing through a 32-gauge hypodermic needle and fractions (0.1 min) were collected by a fraction collector in 1.5-ml polypropylene centrifuge tubes. Fractions were spotted on a reinforced nitrocellulose membrane (Amersham Protran 0.45 µm) in a 96-well suction dot-blot apparatus. The membrane was blocked with BSA and immunostained with p53 phospho-Ser15-specific antibody (Abcam 223868) followed by HRP-linked secondary antibody and imaging of chemiluminescence as described below.

### Western blots

Protein concentration in samples was measured in triplicate by mixing 0.03 ml homogenate with 0.1 ml water + 0.1 ml Coomassie Plus Bradford reagent (ThermoFisher 23238). Bovine serum albumin was used as a standard. Absorbance at 595 nm was measured in a Biotek/Agilent Synergy H1 microplate reader. Samples containing 10–30 µg protein were mixed with 0.5 volume of 5 × non-reducing Laemmli buffer, incubated for 3 min at 95°, and applied to a Novex 4–20% polyacrylamide gradient Tris–glycine wedge gel (Fisher XP04202). In some experiments, a 10% gel poured manually in Mini-Protean short plates (BioRad, 1653308) was used instead. Proteins were electrophoretically transferred to nitrocellulose membranes, blocked for 1 h with 2.5% BSA + 10 mM Tris–HCl pH 8.0 + 150 mM NaCl, incubated in TBST for 1–2 h at RT with primary antibody, and washed 3 times with 1xTBST. Blots were incubated 1 h at RT in TBST with HRP-labeled secondary antibody (0.06 µg), then washed with TBST 6× for 3 min. SuperSignal Atto West chemiluminescence substrate (ThermoFisher, 38555, 1 ml) was added and membranes were imaged at 16 bits in a custom CCD imaging system. In some experiments, Amersham ECL Prime Western Blotting Detection Reagent (RPN2232) was used instead. To confirm equal loading, membranes were stripped by immersion in RestorePlus Western Blot Stripping Buffer (ThermoFisher 46430), blocked with BSA, and stained for actin, GAPDH, or histone H3. Novex LC5800 colored MW markers (ThermoFisher) were used to calibrate the images.

Antibodies used: phospho-S396 IRF3 = Thermo 720012; pan IRF3 = Thermo 582178; STING = Abcam ab239074; phospho-S366 STING = Cell Signaling 197815; 14-3-3σ = Proteintech 10622010ap; pan p53 = Abcam ab26; phospho-S15 p53 = Abcam ab223868; CEBPβ = Santa Cruz sc-7692; γH2AX = Thermo MA533062; GM130 = R&D Systems mab81991-100; actin = ThermoFisher MA515739; GAPDH = ThermoFisher 585074; histone H3 = Abcam ab176842; ATM = Abcam ab32420; pATM = Abcam ab81292; ATR = Abcam ab2905; pATR = Abcam ab223258.

ELISA kits used: IFNα = PBL Assay 41115-1; IFNβ = PBL Assay 41415-1; Protein carbonylation = Abcam ab238536; phospho T1989/total ATR = Abcam ab279742; phospho S1981/total ATM = Abcam ab279740. Ratios obtained from phospho-ELISA kits were corrected for the different signal responses from the pan- and phospho-specific antibodies used by the kit using phosphoprotein standards. To confirm the ELISA results, all ELISA experiments except for interferon measurements were repeated using Western blots. Protein carbonylation was also confirmed by a Protein Carbonyl Assay Kit (Abcam ab178020) in which the proteins are derivatized with dinitrophenylhydrazine, separated by SDS–polyacrylamide gel electrophoresis, and stained with a DNP-specific antibody according to the manufacturer’s instructions.

### Protein carbonylation

Protein carbonylation was measured using the colorimetric Abcam Protein Carbonyl Elisa kit (Abcam ab238536) according to the manufacturer’s instructions. Frozen samples of the temporal lobe obtained from AD and unaffected patients were prepared by homogenization of tissue in 1xPBS containing 1 × HALT protease/phosphatase inhibitors. Samples (0.25 µg protein) were applied to the ELISA plate. Carbonylation Western blots were performed using a Protein Carbonyl Assay Western Blot kit (Abcam ab178020) according to the manufacturer’s instructions. Briefly, 300 µg (around 30 µl) of homogenate was solubilized by combining 30 µl of 2× extraction buffer containing 50 mM dithiothreitol. After a 20 min incubation on ice for 20 min, samples were centrifuged at 18,000×*g* for 20 min at 4 °C. Protein concentration was measured again and aliquots (10 µl) of the solubilized supernatants were denatured with 10 µl of 12% sodium dodecyl sulfate (SDS) for a final concentration of 6% SDS. Samples were derivatized by adding 20 µl of 1× dinitrophenylhydrazine solution and incubated at room temperature for 15 min. Neutralization solution (20 µl) was added and the neutralized samples were applied to a precast 4–20% polyacrylamide gel. Proteins were electrophoretically transferred to nitrocellulose membranes, blocked with bovine serum albumin, and stained using an antibody specific for the DNP moiety in proteins. After 3 × washing with TBST, the membranes were incubated 1 h with LiCor 800CW IRDye anti-rabbit secondary antibody (926-32211) at 1:1000 dilution, washed, and imaged in a custom near-infrared imaging system (excitation = 720–775 nm, emission = 790–810 nm) for 300 s and imaged again for 0.5 s under white light for the MW markers. Images were analyzed densitometrically by scanning horizontally across the image between 30 and 260 kDa.

### Imager and software settings

Chemiluminescent Western blots were imaged at 16 bits/pixel in a custom CCD imaging system with an f/1.2 lens using exposures between 7 and 120 s. Densitometry was done using custom image analysis software (Imal 3.8.5). Background subtraction in dot blots was done by partitioning the original 16 bit grayscale values into foreground and background using the fuzzy k-means algorithm in Imal using sampling of the 40 pixel diameter circular region around the spot. This procedure corrected for any minor variations in background across the blot and was validated against other software before use. For Western blots, a 16 bit/channel image was taken with a red, green, and blue filter showing the colored molecular weight markers on the same blot illuminated by a white LED. The 48-bit RGB color image of MW markers was reduced to 24 bits for publication. The chemiluminescence image was color-inverted, converted to 24 bit grayscale, then to 24 bit RGB, and combined with the MW marker image for publication.

### Statistical analysis

Statistical tests, including two-variable equal variance Student T tests, error propagation, mean and SEM of chromatograms, and Marquardt–Levenberg nonlinear curve fitting, were done in xdata 1.0.0. Experiments in which two or more factors are measured were analyzed by ANOVA followed by Tukey HSD [Honestly Significant Difference] multiple comparisons of means (95% family-wise confidence level) using R. Other statistics, including power analysis, were calculated in R. Data are shown as mean ± standard error of the mean (SEM) unless otherwise indicated.

### Subcellular fractionation

Golgi were isolated using the Invent Biotechnologies Golgi Enrichment Kit (GO-037). This kit was found to produce highly enriched cis-Golgi apparatus and trans-Golgi secretory vesicles as determined by Western blot for Golgi marker GM-130. Tissue was homogenized by grinding 20–50 mg of tissue against the membrane of a filter cartridge with a plastic rod, followed by several centrifugation steps prescribed by the manufacturer. GM-130 is normally dimerized^57^ and 100 mM DTT is necessary to observe the monomeric band. Nuclei for Western blot experiments were isolated as described above for Comet analysis except that the NILR extraction step was done twice and the nuclei were sonicated in a Qsonica Q125 125-Watt sonicator for 20 s at 4° and 20% amplitude after isolation.

## Results

### AD brains exhibit oxidative dsDNA damage but not single-strand DNA (ssDNA) damage

**Figure 1:**
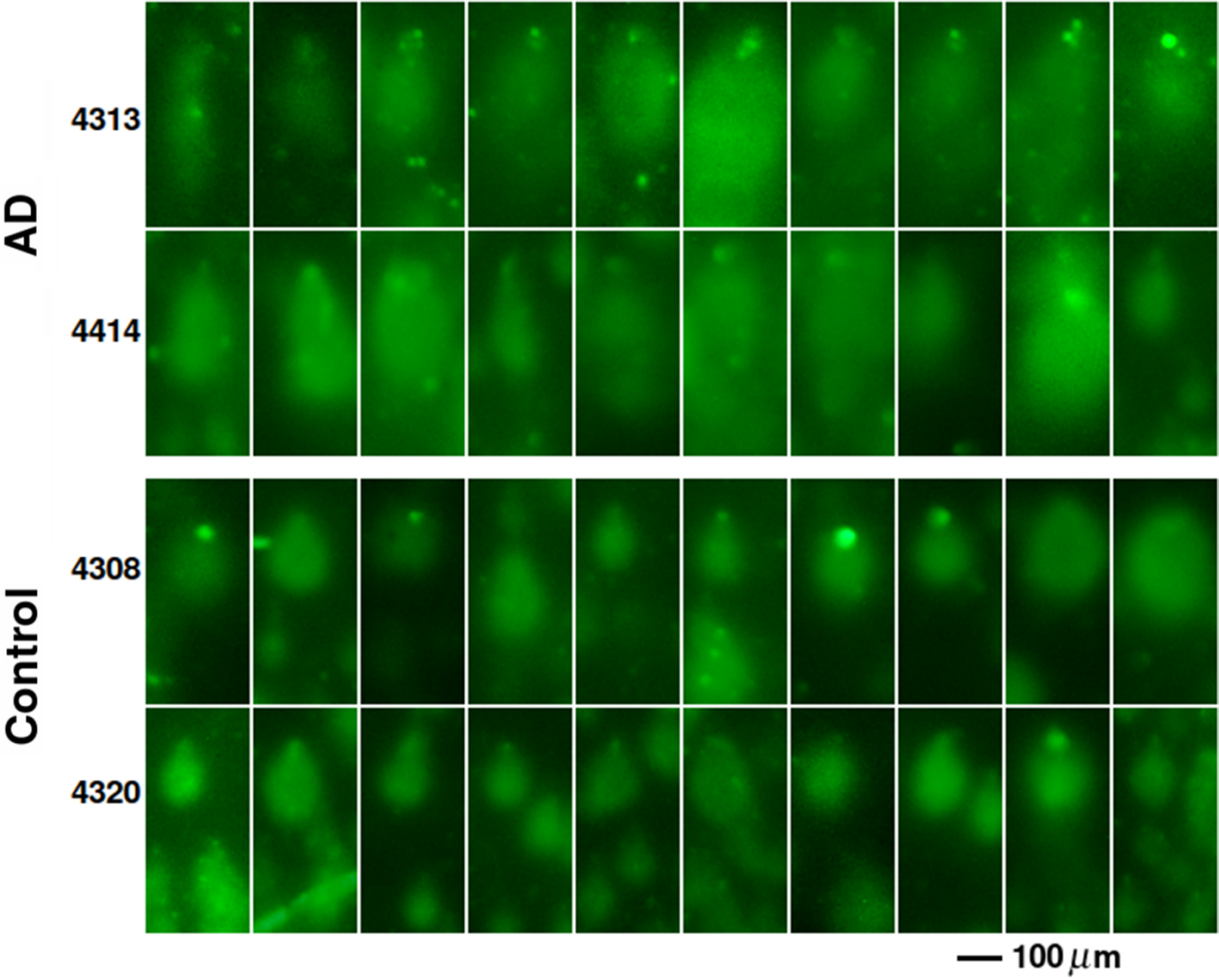
DNA damage is increased in AD. Each panel shows ten randomly selected nuclei isolated from human temporal lobes from two AD and two age-matched control patients. Isolated nuclei were plated onto agarose slides, lysed, and incubated in high-pH unwinding solution. After agarose electrophoresis (4°, 21 V, 1 h), they were stained with SYBR Gold, illuminated with a 402-nm laser, and emission at 530 nm was photographed at a fixed distance from the slide. Representative images are shown. Fifty comets from each image (5 AD and 5 control) were analyzed. Analysis of all ten samples showed that AD samples contained consistently higher percentage of DNA in the tail and increased tail moment (Fraction of DNA in tail: AD = 0.83 ± 0.02; Control = 0.75 ± 0.02, n = 5, p = 0.0091. Tail moment: AD = 1.62 ± 0.16 of control, n = 5, p = 0.0034, two tailed Student’s t test), indicating greater dsDNA breakage in the AD samples.

We measured DNA integrity in nuclei isolated from temporal lobe autopsy samples from 5 AD and 5 unaffected patients using the COMET test. This test allows visualization of DNA damage by subjecting nuclei immobilized in agarose to a low-voltage electric field. The amount of DNA degradation is proportional to the tail moment and length of the ‘comet’ tail. Figure 1 shows that nuclei isolated from AD patients had significantly longer tails than age-matched controls (tail length as tail DNA/total DNA: AD = 0.832 ± 0.018; age-matched control = 0.748 ± 0.017, n = 5, p = 0.0091). Tail moment was also significantly increased (AD = 40.92 ± 3.80; age-matched control = 25.24 ± 0.69, n = 5, p = 0.0034), indicating a higher level of degradation consistent with dsDNA damage or inhibition of DNA repair in AD.

**Figure 2:**
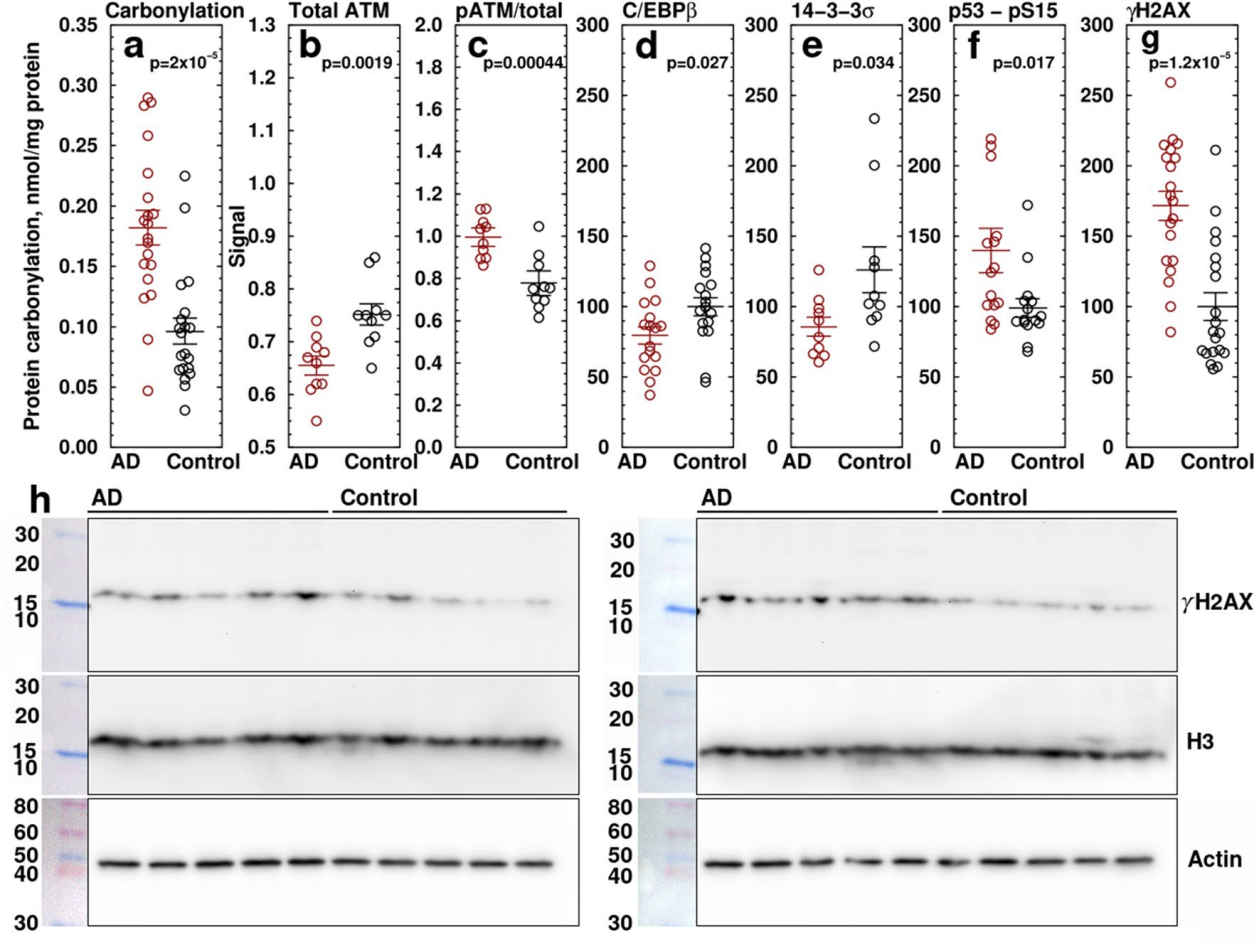
Changes in DNA damage repair-related proteins in brains of AD patients. (**a**) Protein carbonylation measured by ELISA. AD is 1.90 ± 0.26 × control. N = 20, p = 2.5 × 10^−5^. Protein carbonylation was confirmed using the 2,4-dinitrophenylhydrazine Western blot method (Fig. S01l,m). (**b**,**c**) Phospho- and total (pan-) ATM measured by ELISA. (**b**) Total ATM, AD = 0.655 ± 0.0017, Con = 0.752 ± 0.002, p = 0.0019. (**c**) Ratio of phospho-S1981-ATM/pan-ATM. AD = 0.996 ± 0.044, Con = 0.776 ± 0.058, p = 0.00045. (**d**–**h**) DNA repair markers measured by Western blot. (**d**) C/EBPβ normalized to control, AD = 79.53 ± 6.01, Control = 100 ± 6.40, N = 17, p = 0.027. (**e**) 14-3-3σ, AD = 85.6 ± 6.6, Con = 126.1 ± 16. N = 10, p = 0.034. (**f**) phospho-S15 p53 normalized to control, AD = 135.0 ± 12.0, Control = 100 ± 6.6. N = 15, p = 0.017. (**g**) γH2AX, a marker of DSBs, normalized to control, AD = 171.6 ± 10.3, Control = 100 ± 9.76. N = 20, p = 1.2 × 10^–5^. (**h**) Representative Western blot of γH2AX in AD and control samples. The combined γH2AX results are shown in the scatterplot (**f**). Western blots were run and developed in parallel under identical conditions. After imaging, blots were stripped and re-stained with histone H3 and actin. Images shown have been cropped for clarity of presentation. Original full-size blots and confirmatory Western blots of the ELISA results are presented in Supplementary Fig. S01a–s. Samples are temporal lobe, Brodmann’s area 38. Ages: AD = 77.2 ± 1.9 yr, Control = 76.3 ± 1.9 yr. PMI: AD = 14.33 ± 0.73 h, Control = 15.05 ± 0.98 h, N = 20, p = 0.56 (${\overline{\text{x}}}$ ± SEM).

In human AD patients, protein carbonylation, measured by ELISA in samples of temporal lobe (Brodmann’s area 38) obtained at autopsy, was 190 ± 26% that of age-matched unaffected controls (Fig. 2a) (N = 20, p = 2.5 × 10^−5^), indicating elevated oxidative stress in AD. The protein carbonylation changes were confirmed using the DNPH Western blot method (Fig. S01l). Densitometric scans confirmed increased carbonylation in a number of proteins between 30 and 260 kDa (Fig. S01m). Calibration of the Western blot image showed prominent bands at 50, 68, 104, and 155 kDa. This blot also confirms that the oxidized product is proteins and not DNA. However, oxidation of DNA in addition to protein oxidation is not ruled out. Protein carbonylation is an accepted marker of oxidative stress^58–62^. Protein carbonylation in peripheral tissue is observed in obese humans^63^ and in mice fed an obesity-inducing diet^64^, as well as in human aging^65,66^.

DNA damage could be caused by increased oxidative DSBs or by an impairment of the DNA damage response (DDR). To determine whether the DDR is impaired in AD, we used ELISA to measure ataxia telangiectasia mutated (ATM) phosphorylation (S1981), an index of dsDNA repair through the p53 pathway. Pan ATM showed a modest decrease to 87 ± 0.32% of control, while the ratio of pATM/pan ATM was increased to 128 ± 7.8% of control (N = 10, p = 0.00045) (Fig. 2b,c), indicating activation. ATM phosphorylation plays a key role in DDR-induced cell death^11^. Due to possible dephosphorylation in the post-mortem interval, the actual changes in ATM phosphorylation could be larger than observed.

**Figure 3:**
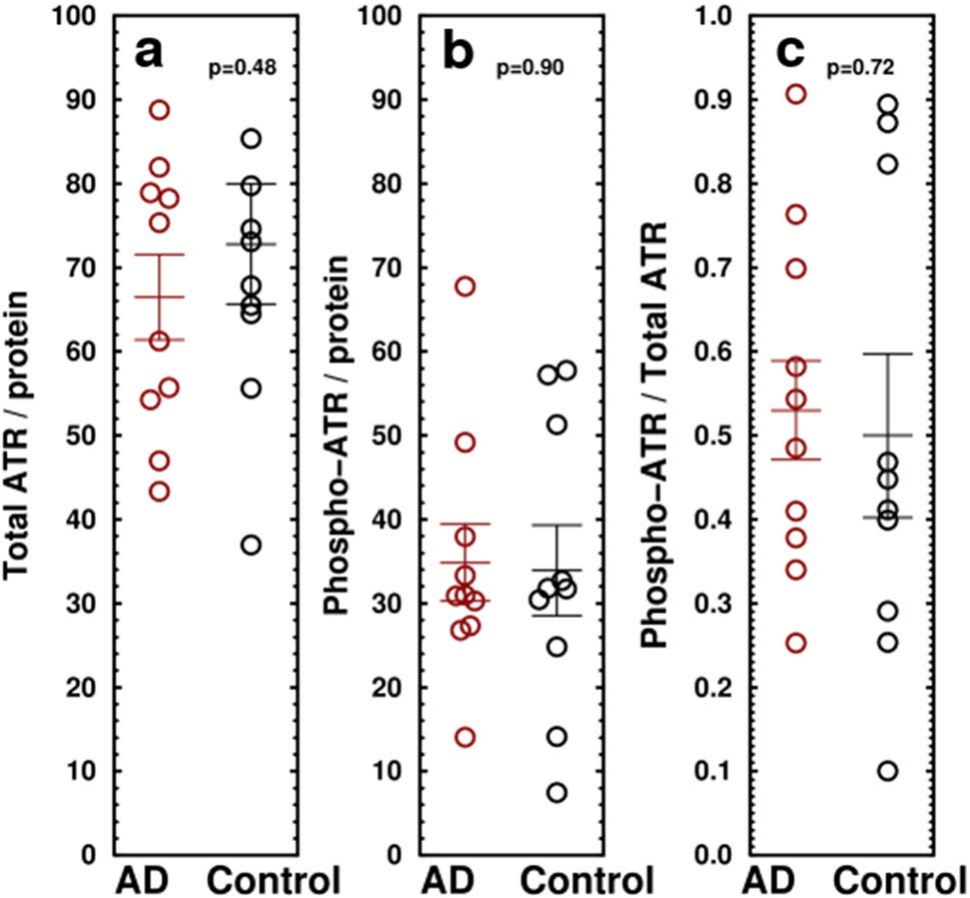
Changes in ATR in AD. Scatterplots show the levels of (**a**) ATR, a marker of ssDNA damage, (**b**) T1989-phosphorylated ATR, and (**c**) ratio of p-ATR to total ATR. No significant changes are evident in temporal lobe of AD patients. Measured by ELISA. This was confirmed by Western blots (Fig. S02).

We also measured C/EBPβ, 14-3-3σ, p53-pS15, and γ-H2AX by Western blotting and ATR and phospho-ATR (T1989) by ELISA. The transcription factor C/EBPβ, a marker of neuronal activity-induced cellular growth, was decreased in AD to 79.5 ± 6.0% of control (N = 17, p = 0.027; Figs. 2d, S01a–k). The 14-3-3σ protein stabilizes the p53 tetramer state by preventing mouse double minute 2 homolog (MDM2) binding^67^, thereby promoting DNA binding^68^. 14-3-3σ was decreased to 67.9 ± 10% of control (Fig. 2e). p53 phosphorylated on S15, an indicator of DNA damage^69^, was elevated to 135 ± 15% in AD samples (AD = 135.0 ± 12.0, Control = 100 ± 6.6, N = 15, p = 0.017 (Fig. 2f). γ-H2AX, a phosphorylated histone marking double strand DNA breaks^70^, despite considerable variability, was 1.72 ± 0.20× of controls (Fig. 2g,h; AD normalized to controls = 171.6 ± 10.3, control = 100 ± 9.8, n = 20, p = 1.2 × 10^–5^). These findings indicate that dsDNA damage is increased in AD. No significant change was observed in ataxia-telangiectasia and Rad3-related protein (ATR), a marker for repair of ssDNA breakage (Fig. 3a), or in its phosphorylation state (Fig. 3b,c) indicating that increased DSBs and dsDNA repair but not ssDNA repair is detectable in AD. The ELISA results for pATM and pATR were confirmed by Western blotting (Figs. S01n,o, S02). Because γ-H2AX is partially localized to nuclei, both histone H3 and actin were used as loading controls (Figs. 2h, S01k).

### p53 oligomerization is compromised in AD

**Figure 4:**
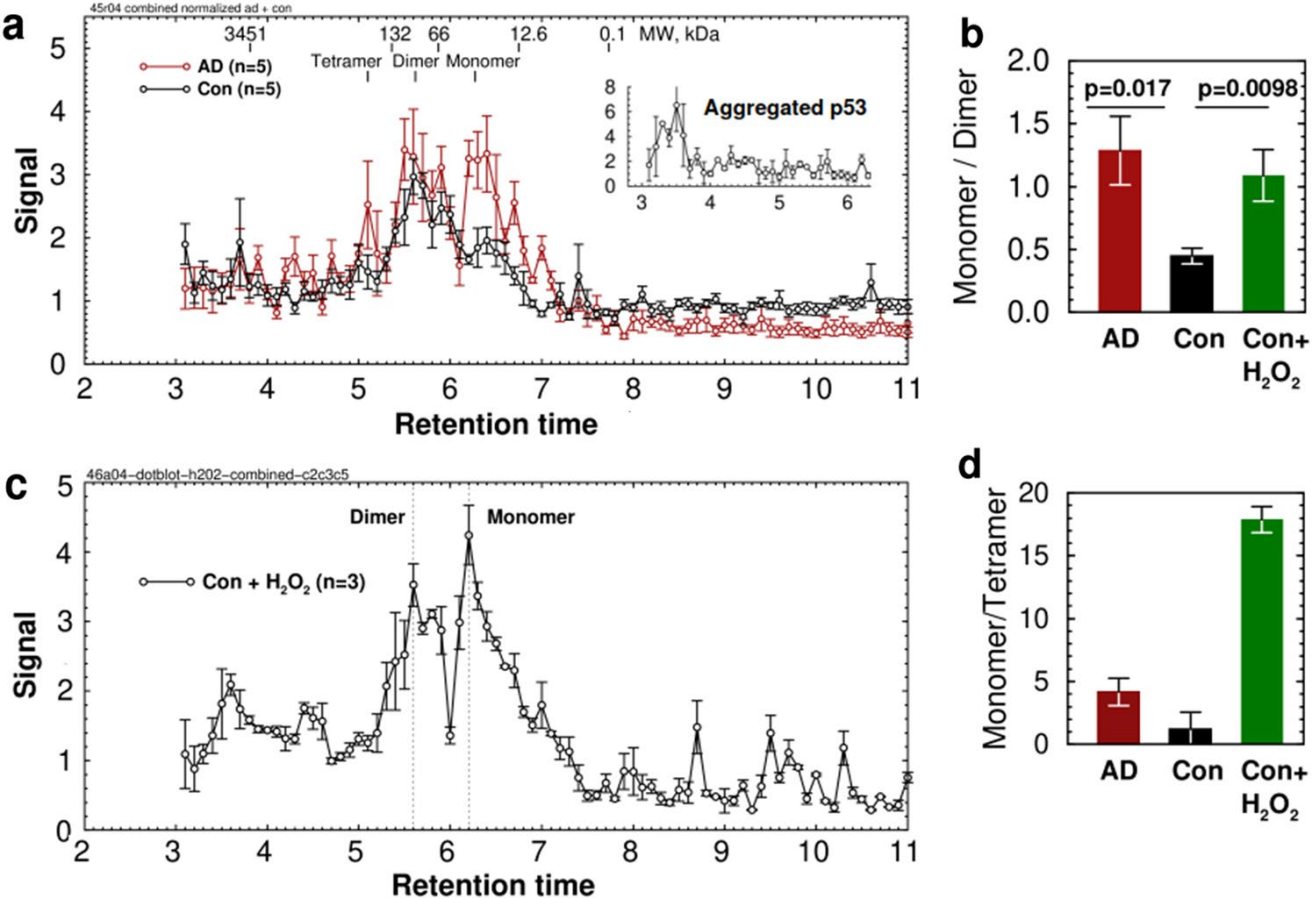
Phosphorylated (S15) p53 oligomerization state is changed in AD. Extracts from human AD and control temporal lobe were chromatographed on size-exclusion HPLC and analyzed by p53-pS15 dot blot followed by densitometry. (**a**) Averaged chromatograms from temporal lobe of five AD and five age-matched controls. Inset shows a chromatogram from a sample that was deliberately concentrated to induce unfolded p53 and aggregation. (**b**) Ratio of monomer/dimer peak areas calculated from the chromatograms. AD = 1.29 ± 0.27, Con = 0.45 ± 0.06, Con + H_2_O_2_ = 1.09 ± 0.20, ${\overline{\text{x}}}$ ± SEM. n = 5, AD vs. Con: p = 0.017, N = 5. (**c**) Oxidation by 100 nM hydrogen peroxide for 30 min increases p53 monomer/dimer ratio. (**d**) Ratio of monomer/tetramer peak areas calculated from the chromatograms. AD = 4.17 ± 1.10, Con = 1.22 ± 1.35, Con + H_2_O_2_ = 17.9 ± 1.0, n = 3, n.s.

Although changes in p53, including unfolding and aggregation in lymphocytes, have been found to occur in AD, measurements of total p53 show modest and sometimes conflicting results, likely due to rapid proteasomal degradation. Changes in p53 oligomerization state in AD have not been reported. We homogenized temporal lobe autopsy samples from AD and age-matched unaffected patients at high dilution (1:50) to avoid artifactual aggregation and measured the oligomerization state of phospho(S15)-p53 in 15,000×*g* extracts using size-exclusion HPLC. Samples (0.1 min) were collected and p-p53 was measured by densitometric analysis of dot blots stained with a phospho(S15)-p53 specific antibody. Nutlin-3, an inhibitor of MDM2 (Kd = 90 nM; 300 nM used), and phosphatase/protease inhibitors were present throughout the procedure to minimize artifactual changes. The results showed a marked increase in monomeric p-p53 in AD samples compared to unaffected patients. Small amounts of tetrameric p53 in cytosol were also detectable in AD samples but not controls (Peak areas: Tetramer AD = 3.66 ± 1.41, Con = 0.95 ± 0.52, p = 0.11. Dimer: AD = 9.15 ± 2.11, Con = 6.63 ± 0.78, p = 0.29. Monomer: AD = 9.92 ± 1.73, Con = 2.99 ± 0.57, p = 0.0052; ${\overline{\text{x}}}$ ± SE, n = 5; Fig. 4a). These differences were not due to the presence or formation of aggregated p53, which elutes at 3.5 min (Fig. 4a inset). These results are consistent with previous studies that found that only 30% of p53 in healthy patients is monomeric^71^. However, the increased monomer fraction in AD was contrary to expectation. Native unmodified monomeric p53 tetramerizes spontaneously^72,73^. Thus, a change in oligomerization state indicates a stable structural change in p53. The monomer:dimer ratio in AD samples was 2.86 ± 0.72 × Control, N = 5, p = 0.017 (Fig. 4b), while the monomer:tetramer ratio showed no statistically significant change (Fig. 4d). Only small levels of p-p53 were detected in isolated nuclei, and no differences in nuclear p-p53 were observed on Western blots (Fig. S03), indicating that the result is not due to changes in tetramerized p53.

Incubation of control temporal lobe homogenates for 30 min with 100 nM H_2_O_2_ increased the monomer:dimer ratio nearly to AD levels (Fig. 4c). These are physiologically attainable levels: inflammation can raise H_2_O_2_ levels in neurons by 25–75x, reaching 66 nM in the case of respiratory chain stimulation by insulin in cerebellar granule neurons^74^. Thus, p53 protein oxidation is a possible explanation for the increased monomer/dimer ratio in AD. However, p53 exists in at least 9 isoforms produced by alternative splicing and is potentially subject to a large number of other post-translational modifications including ubiquitination, phosphorylation, acetylation, and methylation. We cannot rule out the possibility that these forms of p53 also exhibit differential tendencies for oligomerization.

### STING is depleted from Golgi in AD

**Figure 5:**
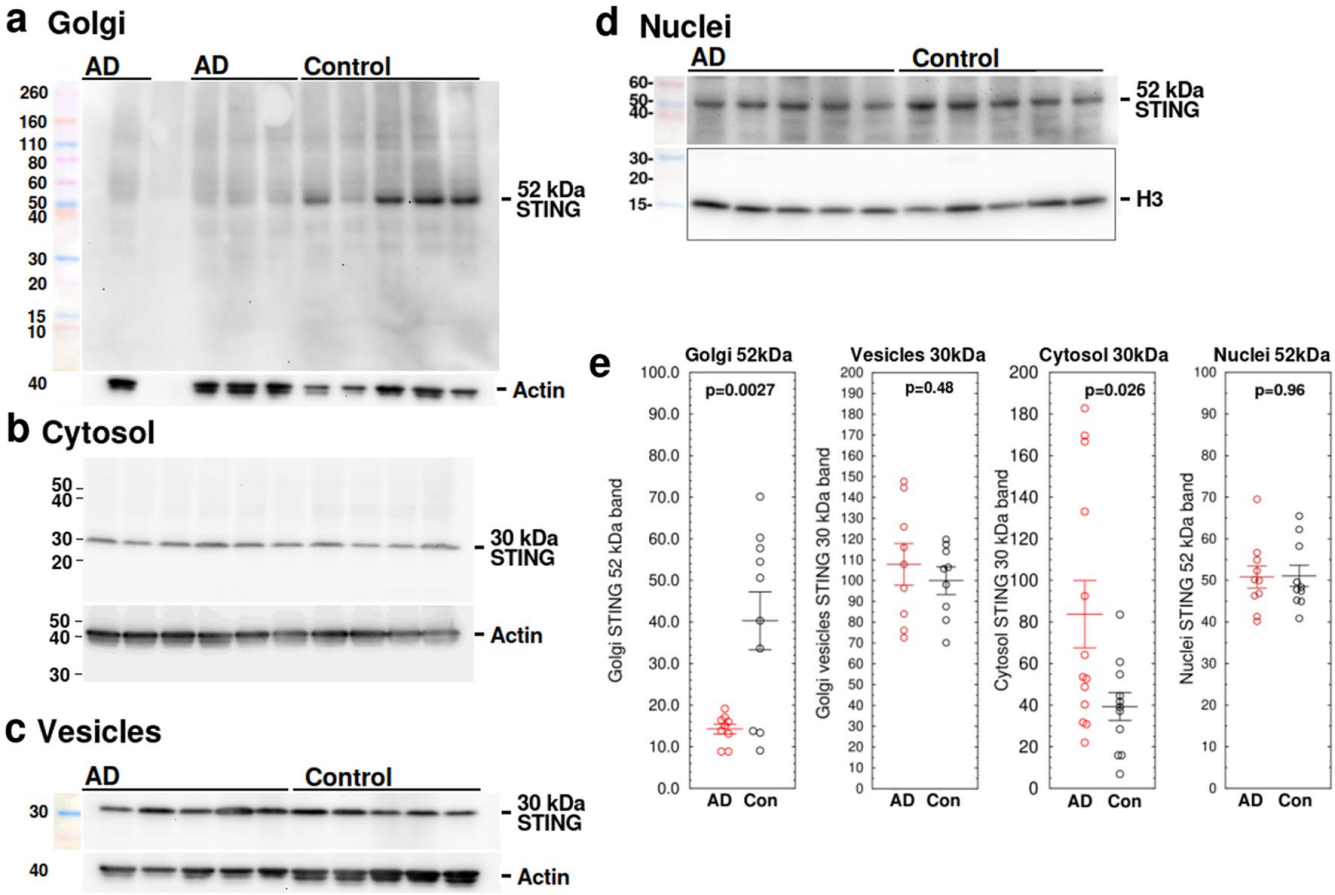
STING protein is depleted from Golgi in AD. (**a**) Representative Western blots of the 52 kDa isoform of STING in enriched Golgi fractions from AD and control temporal lobe, measured by densitometry. STING was 2.82 ± 0.54 × lower in AD patients than controls (AD = 14.3 ± 1.18, control = 40.3 ± 6.9, ${\overline{\text{x}}}$ ± SEM, n = 10, p = 0.0027). (**b**) The 30 kDa isoform of STING was moderately increased in cytosol extracted from the same samples (AD = 83.7 ± 16.2, control = 39.4 ± 6.7, n = 13, p = 0.026, ${\overline{\text{x}}}$ ± SEM). (**c**) Representative Western blot of 30 kDa isoform of STING protein from isolated Golgi vesicles from AD and control temporal lobe. No statistically significant change was observed. (**d**) Representative Western blot of 52 kDa isoform of STING protein from isolated nuclei from AD and control temporal lobe. No statistically significant change was observed. (**e**) Scatterplots of combined data from Western blots of 52 kDa and 30 kDa STING protein in Golgi, cytosol, and nuclei. Western blots in (**a**–**c**) and d are different blots. Blots were stripped and re-probed with actin (**a**–**c**) or histone H3 (**d**). Colored MW markers were photographed on the corresponding blot at the time of initial immunostaining. Western blot images shown have been cropped for clarity of presentation. Original full-size blots are presented in Supplementary Fig. S04a–n. Scatterplots represent combined densitometry results from 10 to 13 AD samples and an equal number of age-matched unaffected patients (Con).

Cytosolic DNA fragments are potent inducers of interferon expression via the STING-cGAS pathway. STING protein exists in several alternatively-spliced forms^75^. In human brain, the 52 kDa wild-type and 30 kDa Isoform 1 are highly abundant. The 52 kDa isoform is translocated to Golgi in response to elevated cGAMP produced by cGAS (cGAMP synthase) after DNA damage, while the 30 kDa isoform is found only in cytosol. To determine whether the damaged DNA in AD activates the STING pathway, we used the Minute Golgi Apparatus Enrichment Kit (Invent Biotechnologies, Plymouth MN) to enrich Golgi apparatus and Golgi secretory vesicles from autopsy samples from patients with AD and controls. While little change was seen in 52 kDa and 30 kDa forms of STING in raw homogenates, cytosol, or nuclei, 52 kDa wild-type STING in Golgi fractions measured by densitometry was 2.82 ± 0.54 × lower from AD patients than controls (AD = 14.3 ± 1.18, control = 40.3 ± 6.9, ${\overline{\text{x}}}$ ± SE, n = 10, p = 0.0027), suggesting almost total depletion of STING in Golgi in AD (Fig. 5a). The 30 kDa cytosolic isoform was also increased in AD samples to 213 ± 55% control (p = 0.026, n = 13, Fig. 5b). No change in the 30 kDa isoform of STING was observed in secretory vesicles (Fig. 5c) and no change in the 52 kDa isoform of STING was observed in nuclei (AD = 50.8 ± 2.7, control = 51.1 ± 2.6, ${\overline{\text{x}}}$ ± SE, n = 10, p = 0.96; Figs. 5d,e, S04).

**Figure 6:**
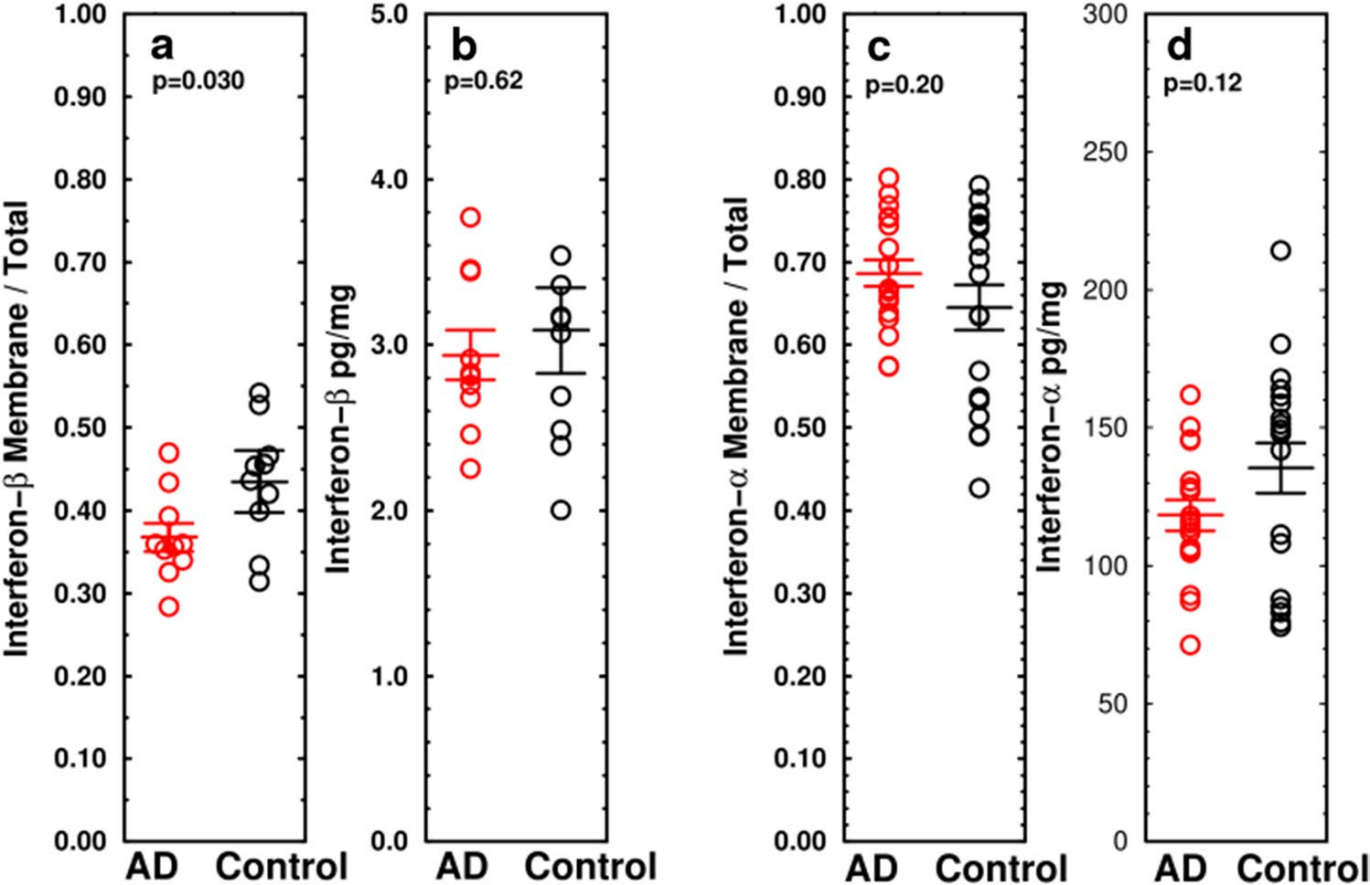
Total and membrane-associated interferon α and β measured by ELISA show little or no change in AD compared to control temporal lobe samples. (**a**,**b**) Membrane-associated IFNβ is moderately decreased in AD (ratio of particulate/cytosol: AD = 0.592 ± 0.045 pg/mg protein, control = 0.797 ± 0.073, p = 0.030, n = 10/group, ${\overline{\text{x}}}$ ± SEM) while total IFNβ shows no effect in AD. (**c**,**d**) Membrane-associated IFNα and total IFNα do not differ between AD and unaffected patients.

Consistent with this finding, the fraction of interferon-beta (IFNβ) in particulate fractions (presumed to represent predominantly receptor-bound IFN), measured by ELISA, was slightly decreased in AD (AD = 0.368 ± 0.017, Con = 0.435 ± 0.037, n = 10, p = 0.030) while IFNβ in cytosol was not affected in AD (2.94 ± 0.15, Con = 3.09 ± 0.26 pg/mg protein, n = 10, p = 0.62) (Fig. 6a,b). Total IFNα and membrane/total IFNα were not affected by AD (Fig. 6c,d), confirming a failure of STING to produce a significant interferon response to DNA damage. This finding is consistent with the observed depletion of wild-type 52 kDa STING from Golgi in AD samples. These results suggest that the STING-IFN pathway is compromised in AD.

### IRF3 phosphorylation is decreased in AD

**Figure 7:**
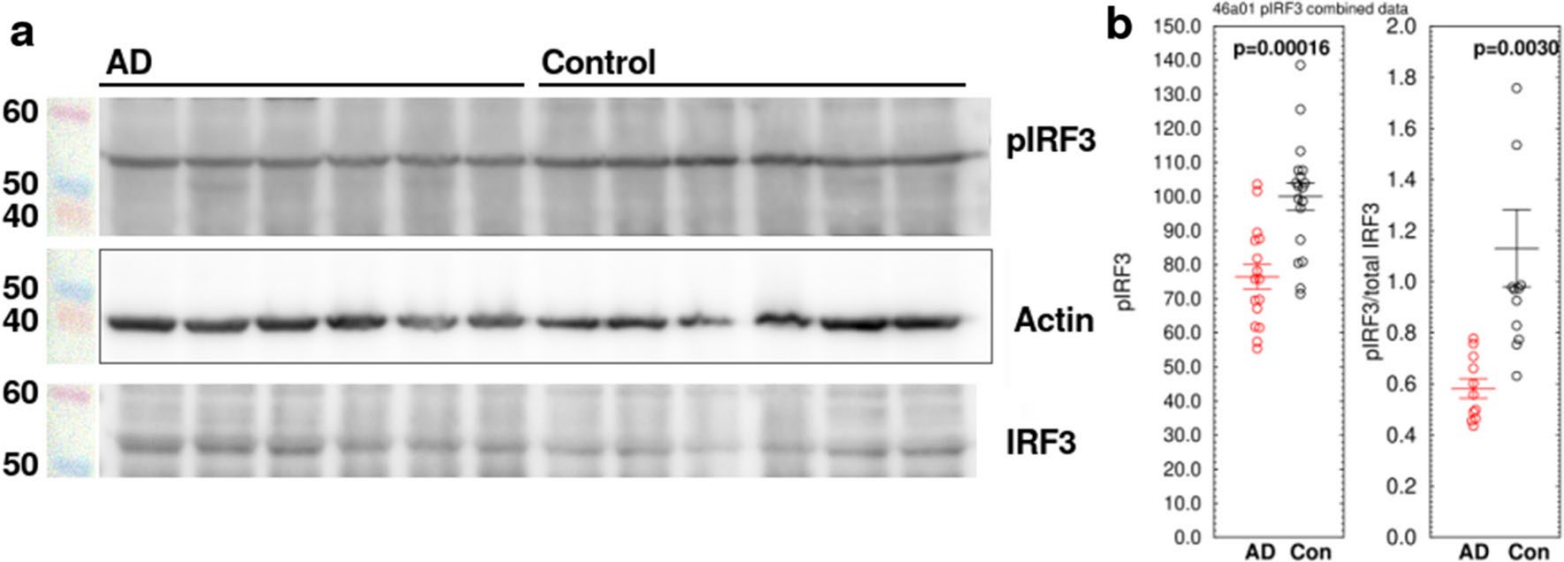
Phosphorylation of IRF3 is decreased in homogenates of AD temporal lobes compared to age-matched controls. (**a**) Representative Western blot. (**b**) Scatterplots of all pIRF3 samples measured by densitometry of Western blots. IRF3 phosphorylation (S396) was decreased in AD to 76.5 ± 4.5% of control (n = 18, p = 0.00016). The ratio of pIRF3/total IRF3 was also decreased (AD = 0.582 ± 0.038, control = 1.131 ± 0.152, n = 11, p = 0.0030), suggesting that phosphorylation of IRF3, which is required for formation of the trimeric complex, is inhibited in AD. All three panels are from the same blot. Western blot images shown have been cropped for clarity of presentation. Original full-size blots are presented in Supplementary Fig. S05. After imaging, the pIRF3 blot in (**a**) was stripped and re-probed for actin, then re-probed for total IRF3. Colored MW markers were photographed on the blot at the time of pIRF3 staining. Scatterplots represent combined densitometry results from 11 to 18 AD samples and an equal number of age-matched unaffected patients (Con).

There is evidence that the p53 and STING pathways are interdependent. p53 knockdown prevents a type I interferon-mediated immune response to influenza virus^76^, and cancer cells with constitutively inactive p53 have deficiencies in cGAS-STING signaling^77^. In cancer cells, inactive mutant p53 binds to TBK1 (TANK-binding kinase), blocking the phosphorylation of IRF3 (interferon regulatory factor 3) by TBK1 and suppressing the formation of the STING-TBK1-IRF3 trimeric complex required for interferon expression^77^. This suggests a mechanism by which the increase in p53 monomerization in AD could produce the effects on STING and interferon that we observed. Western blots (Fig. 7) showed that IRF3 phosphorylation was decreased in AD to 76.5 ± 4.5% of control (n = 18, p = 0.00016). The ratio of pIRF3/total IRF3 was also decreased (Figs. 7, S05) (AD = 0.582 ± 0.038, control = 1.131 ± 0.152, n = 11, p = 0.0030), suggesting that phosphorylation of IRF3, which is required for formation of the trimeric complex, may be inhibited in AD. Further experimentation is needed to determine whether p53 monomerization alone is sufficient to inhibit TBK1 signaling and to establish the mechanism by which p53 isoforms and post-translational modifications and oligomerization state inhibit IRF3 phosphorylation.

## Discussion

These results provide evidence that the DNA damage repair (DDR) response is compromised in AD. Oxidation of p53 by ROS could affect the DDR by altering the oligomerization state of p53. Unrepaired DSBs result in somatic mutations and other catastrophic effects for a cell. Evidence suggests that the DSBs are produced in part by ROS created during inflammation by activation of non-mitochondrial^78^ NADPH oxidase (NOX), which is normally inert but is activated by cytokines, angiotensin II, hypoxia, or oxidized fatty acids which are found in obesity^79–82^. Overactivation of NOX in cerebrovascular endothelial cells is one of the main causes of cerebrovascular damage in AD^83^. Apocynin, a NOX inhibitor, reduces vascular β-amyloid deposits by 80% in patients with cerebral amyloid angiopathy (CAA)^84^, a co-morbidity in > 90% of AD patients. Thus, it is possible that much of the unrepaired DNA damage could be attributed to NOX activated in patients carrying risk factors for AD, including inflammation and obesity.

Many links have been reported between DNA damage and tau neurofibrillary tangles (NFTs), which are a hallmark of AD. Extensive DNA damage as shown by the terminal deoxynucleotidyl transferase-dUTP nick end labeling (TUNEL) assay occurs in cerebral cortical neurons in AD^85,86^. This DNA damage is associated with NFTs^87^ and has been attributed to neuronal vulnerability and necrosis rather than apoptosis^88,89^, although some researchers also report apoptotic morphologies^90^. NFTs contain phospho-tau, an established biomarker for AD, and tauopathy may be a driver for neuron loss in AD via necroptosis, possibly involving cofactors such as Transactive Response DNA-binding protein 43 (TDP-43) and Aβ^91^. Co-expression of 3R-tau isoforms can induce oxidative DSBs along with glial activation, neuronal death, and memory deficits^92^. These effects are reversed by antioxidants^92^. Tau can also interact with p53: depletion of tau in cultured neuroblastoma cells alters p53 stability and reduces cell death^93,94^. DSBs and phospho-tau frequently co-localize in AD cortex. Knockdown of tau exacerbates DSBs in neurons, suggesting a role for tau in DNA repair^95^, consistent with a role for tau in protecting neuronal genomic DNA integrity^96^.

Links have also been reported between Aβ plaques and DNA damage. Aβ induces oxidative stress, in part by interfering with normal mitochondrial activity^97^, and induces oxidative DNA damage^79^ as shown by 8-hydroxy-2'-deoxyguanosine (8-OHdG) staining and histone phosphorylation (γ-H2AX)^98–100^. Aβ may also contribute indirectly by inhibiting NHEJ by blocking DNA-dependent protein kinase, thereby promoting oxidative DNA damage^101^. Genomic DNA damage is reported to induce neuronal production of Aβ by increasing β-secretase activity^102^. Besides immunotherapy to deplete soluble Aβ and plaques^103^, a variety of treatments have been proposed to inhibit these toxic effects of Aβ, including glutamine^104^, galantamine^105^, antioxidants^106^, and isoflavones^107^. Lesions in myelinating oligodendrocytes in gray matter^108^ and damage to astrocytes^109^ also correlate with DNA damage, suggesting that DNA damage in non-neuronal cells may also contribute to AD. However, cognitively healthy centenarians have NIA (amyloid) and Braak (NFT) stages similar to AD patients, suggesting that a portion of the population is cognitively resilient to AD^110,111^.

The results also provide evidence that the cGAS-STING pathway, which is activated by pathogen-derived and self-DNA, mediates immune surveillance, and induces the Type I interferon response, is impaired in the temporal lobe of human patients with AD despite evidence of DSBs. Our finding that IRF3 phosphorylation is decreased in AD, combined with our finding that oligomerization of p53 is inhibited in AD, suggests that a similar mechanism could explain the observed effects on STING and interferon. However, further experimentation is necessary.

**Figure 8:**
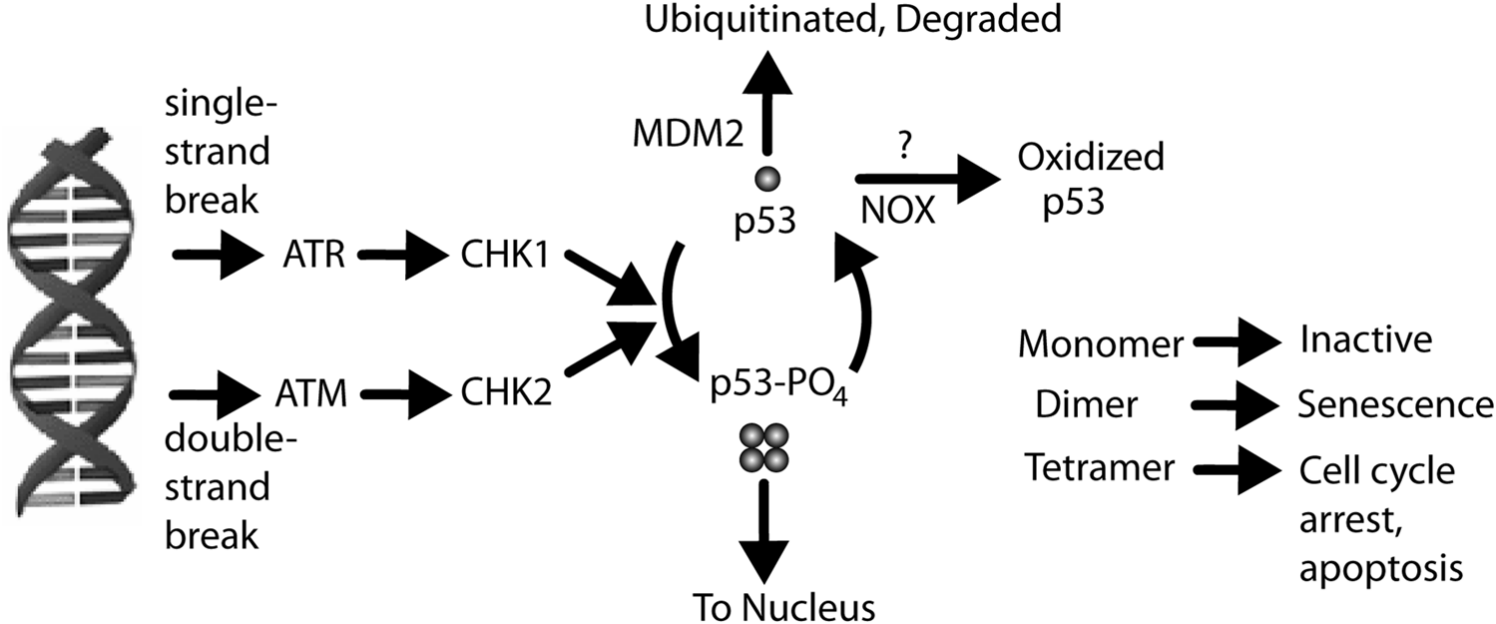
Role of p53 in DNA repair pathway. The DNA damage repair pathway involves activation of ATR for ssDNA breaks and ATM for dsDNA breaks. These kinases act on CHK1 and CHK2 to phosphorylate p53. Dephosphorylated p53 is ubiquitinated by MDM2 and rapidly degraded at the proteasome. Phosphorylation of p53 at S15 inhibits interaction with MDM2. p53 induces MDM2 transcription, ensuring that the lifetime of p53 is short. Phosphorylation and oligomerization state determine p53’s effect so that p53 acts as a switch to control the cell’s response to DNA damage. The tetramerized form most strongly binds DNA. Many other post-translational modifications of p53 have been described.

Whether p53 induces cellular senescence or apoptosis depends on its oligomerization state (Fig. 8) and a large number of potential post-translational modifications^112–117^. Phosphorylation of p53 at S15 leads to decreased interaction with its negative regulator, MDM2^13^, an E3 ubiquitin kinase that targets p53 for proteasomal degradation, leading to higher levels of p53 protein^118,119^. Phosphorylation at S15 and S20 increases p53 stability, enhances tetramerization^120^, and favors senescence, while phosphorylation at S46 combined with acetylation at K382 induces apoptosis^121,122^. Conversely, NOX1 (NADPH oxidase 1) impairs p53 K382 acetylation, preventing apoptosis^123^. ROS produced during inflammation could induce the dissociation of dimeric and tetrameric p53 to the inactive monomer form and thereby inhibit DNA repair and interferon signaling. If so, shifting the redox balance could restore p53 to its active state and thereby enhance DNA repair. As mentioned above, Aβ can also induce oxidative stress, which induces DSBs. Aβ was also reported to increase expression of p53 and its transcription target, Bax, in cultured cortical neurons^124^. Upregulation of p53 by Nutlin-3 is reported to prevent Aβ42-induced DNA damage^125^. However, other studies report that inactivation of p53 has no effect on induction of apoptosis by Aβ(25–35) in cultured cells, suggesting that Aβ-induced apoptosis does not depend on p53.

The finding of impaired p53 oligomerization in AD reported here is entirely distinct from p53 misfolding or unfolding. Misfolded p53 forms amyloid-like aggregates that impair its DNA-binding function^43,126^. Misfolded p53 has been found in PBMCs^127,128^ and fibroblasts^129^ but not neurons^130^ and has been proposed as an early marker of AD. Misfolded p53 is easily measured and elutes at 3.5 min in SEC-HPLC (Fig. 6a, insert). However, no non-artifactual unfolded p53 was observed in our CNS samples and there are no reports of it in CNS. Unfolding of p53 is unrelated to its oligomerization state^131^ and can be produced when p53 is not bound to its cognate DNA^132^ or when p53 is artificially concentrated in vitro*.* The involvement of p53 also provides a possible explanation for the long-hypothesized inverse connections between AD and cancer, recently replicated in large longitudinal studies^133–135^.

While cGAS is normally thought of as an activator of STING, chronic activation of cGAS by persistent unrepaired DNA damage can also induce STING to undergo a phase transition to a membranous condensate that isolates STING from its effector protein IRF3^136,137^. Thus, depletion of STING from Golgi in AD patients, and hence loss of interferon responses, could be a response to chronic DNA damage. The cortex is composed primarily of neurons, and we cannot rule out a similar or compensatory effect in glial cells. Impaired interferon responses would increase the risk of AD progression^138^ and drive microglial dysfunction^139^.

Antioxidants have been proposed many times as general nonspecific treatments for oxidative stress in AD. However, designing effective antioxidant therapies specific for AD requires knowledge of specific targets and mechanisms in AD. p53 activity and tetramerization are strongly dependent on redox factor 1 (Ref-1/APE1), a protein involved in base excision repair, and thioredoxin, which reactivates oxidized p53^140–143^. Thus, elevating thioredoxin levels could be another potential way of restoring DNA repair in AD.

## Supplementary Information

Supplementary Information.

## Data Availability

The datasets generated during and/or analyzed during the current study are available from the corresponding author on reasonable request.

## Code availability

Source code and related files for the software used in data analysis are available from the corresponding author on reasonable request.
