# Supplementary material for: Sting and p53 DNA repair pathways are compromised in Alzheimer’s disease
Source: Sci Rep. 2023 May 23;13:8304. doi: 10.1038/s41598-023-35533-6 (PMC10206146; doi:10.1038/s41598-023-35533-6)
Supplement: Supplementary file 1 — Supplementary Information. [file 41598_2023_35533_MOESM1_ESM.pdf]

Supplementary Data

Sting and p53 DNA Repair Pathways Are Compromised in Alzheimer's Disease

Thomas J. Nelson and Yunhui Xu

SUPPLEMENTARY FIGURES

In each Western blot figure, the first five lanes are from AD samples and the last five lanes are from controls unless indicated on the figure.

Fig. S01

Fig. S01a Western blot of H2AX in AD temporal lobe samples. 120s exposure.

Fig. S01b Western blot of H2AX in control temporal lobe samples. 120s exposure.

Fig. S01c Western blot of actin loading control in H2AX AD samples. 120s exposure.

Fig. S01d. Western blot of actin loading control in H2AX control temporal lobe samples. 120s exposure.

Fig. S01e. Western blot of H2AX in 5 AD (lanes 1 – 5) and 5 control (lanes 6 – 10) temporal lobe samples. 30s exposure.

Fig. S01f. Western blot of H2AX in 5 additional AD (lanes 1 – 5) and 5 additional control (lanes 6 – 10) temporal lobe samples. 30s exposure.

Fig. S01g. Western blot of histone H3 loading control for Fig. S01e. The blot was stripped and re-stained for H3, which migrates at a slightly lower MW than H2AX.

Fig. S01h. Western blot of histone H3 loading control for Fig. S01f. The blot was stripped and re-stained for H3, which migrates at a slightly lower MW than H2AX.

Fig. S01i. Visible light image of blot imaged in Fig. S01e and S01g showing molecular weight markers.

Fig. S01j. Visible light image of blot imaged in Fig. S01f and S01h showing molecular weight markers.

Fig. S01k. Composite of H2AX Western blots from Fig. S01e – S01h with grayscale values inverted to show bands as black on white background. These blots were run and developed under identical conditions.

Fig. S01l. Western blot of protein carbonylation in 5 AD and 5 Control temporal lobe samples treated with 2,4-dinitrophenylhydrazine and stained with DNP-specific antibody. Secondary antibody was LiCor 800CW IRDye and the blot was imaged at 790 – 810 nm near infrared light. This blot confirms the ELISA results in Fig. 2a and also confirms that the oxidized product is proteins and not DNA. However, oxidation of DNA in addition to protein oxidation is not ruled out. Calibration of the image shows prominent bands at 50, 68, 104, and 155 kDa.

Fig. S01m. Densitometry tracing of the DNP protein carbonylation Western blot in Fig. S01l. Image was scanned horizontally at the region across the entire blot between 30 kDa and 260 kDa and baseline was subtracted automatically. This corrects for any variation in the background and confirms that protein carbonylation is elevated in AD temporal lobes.

Fig. S01n. Western blot of phospho-ATM in AD and Control temporal lobes. This blot confirms the ELISA results in Fig. 2c showing increased phosphorylation of ATM in AD.

Fig. S01o. Western blot of actin staining as loading control for pATM in Fig. S01n (120 sec exposure) . Blot was stripped and re-probed for actin.

Fig. S01p. Western blot of actin staining as loading control for γH2AX Fig. S01e (10 sec exposure) . After s staining for histone H3, blot was stripped again and re-probed for actin. Lanes 1 – 5 are AD, lanes 6 – 10 are Controls.

Fig. S01q. Western blot of actin staining as loading control for γH2AX Fig. S01f (10 sec exposure) . After staining for histone H3, blot was stripped again and re-probed for actin. Lanes 1 – 5 are AD, lanes 6 – 10 are Controls.

Fig. S01r. Western blot of actin blot in Fig. S01p exposed for 480 sec to show edges of membrane.

Fig. S01s. Western blot of actin blot in Fig. S01q exposed for 480 sec to show edges of membrane.

Fig. S02

Fig. S02. Western blot of phospho-ATR in AD and control temporal lobe autopsy samples. No change is evident. This confirms the ELISA results in Fig. 3c.

Fig. S03

Fig. S03. Western blot of phospho(S15)-p15 showing no change in p53 in nuclei. Very faint staining is observed. Image was inverted so bands appear black instead of white. 60s exposure.

Fig. S04

Fig. S04a. Western blot of 52 kDa isoform of STING protein in isolated Golgi. 300s exposure.

Fig. S04b. Western blot of actin in isolated Golgi as loading control for Fig. S04a. 60s exposure.

Fig. S04c. Western blot of STING protein in isolated Golgi secretory vesicles. 60s exposure. Only the 30 kDa isoform was seen. No significant change was found. See Fig. S04m for longer exposure.

Fig. S04d. Western blot of actin in Golgi secretory vesicles. 120s exposure.

Fig. S04e. Western blot of STING protein in cytosol. 120s exposure. The principal band is at 30 kDa, although the 52 kDa isoform is visible in the control samples.

Fig. S04f. Western blot of actin for fig. S04e. Blot was stripped and re-probed for actin. 90s exposure. See Fig. S04n for longer exposure.

Fig. S04g. Western blot of STING protein in isolated nuclei. The signal is very faint even after a 1440 second exposure shown to illustrate the bands and membrane boundary.

Fig. S04h. Western blot of H3 loading controls in nuclei. 20s exposure. See Fig. S04l for longer exposure.

Fig. S04i. RGB image of blot of isolated Golgi vesicles showing MW markers.

Fig. S04j. Western blot of STING protein in isolated nuclei. Image grayscale values have been inverted and matching RGB lane from the same blot has been superimposed on the image to show molecular weight markers.

Fig. S04k. Western blot of H3 gel loading controls in isolated nuclei. Same blot as S04h except image grayscale values have been inverted and matching RGB lane from the same blot has been superimposed on the image to show molecular weight markers.

Fig. S04l. Western blot of H3 gel loading controls in isolated nuclei. Same blot as S04k except 160s exposure to show edges of membrane. All H3 bands are saturated at 65535 in this image.

Fig. S04m. Western blot of STING in Golgi secretory vesicles. Same blot as Fig. S04c except 240s exposure to show edges of membrane. STING bands are saturated at 65535 in this image.

Fig. S04n. Western blot of actin for fig. S04e. Same blot as Fig. S04f except 960s exposure to show edges of membrane. Actin bands are saturated at 65535 in this image.

Fig. S05

Fig. S05a. Western blot of phospho-IRF3 in AD and control homogenate. 10s exposure

Fig. S05b Western blot of actin in AD and control homogenate (stripped and re-probed from S05a). 30s exposure. See Fig. S05d for longer exposure

Fig. S05c. Western blot of pan-IRF3 in AD and control homogenate (stripped and re-probed from S05b). 10s exposure. . Faint staining is evident. Cross-reactivity is evident in all blots from this antibody

Fig. S05d Western blot of actin (stripped and re-probed from S05a). Same blot as S05b except 240s exposure

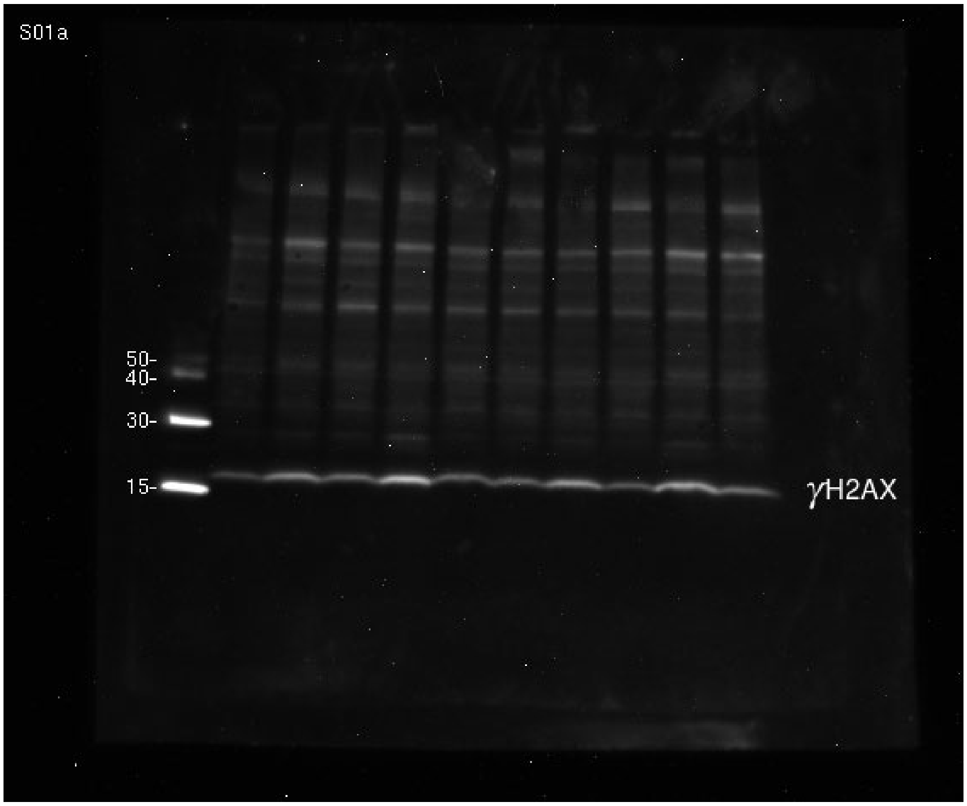
S01a
50-
40-
30-
15-
γH2AX

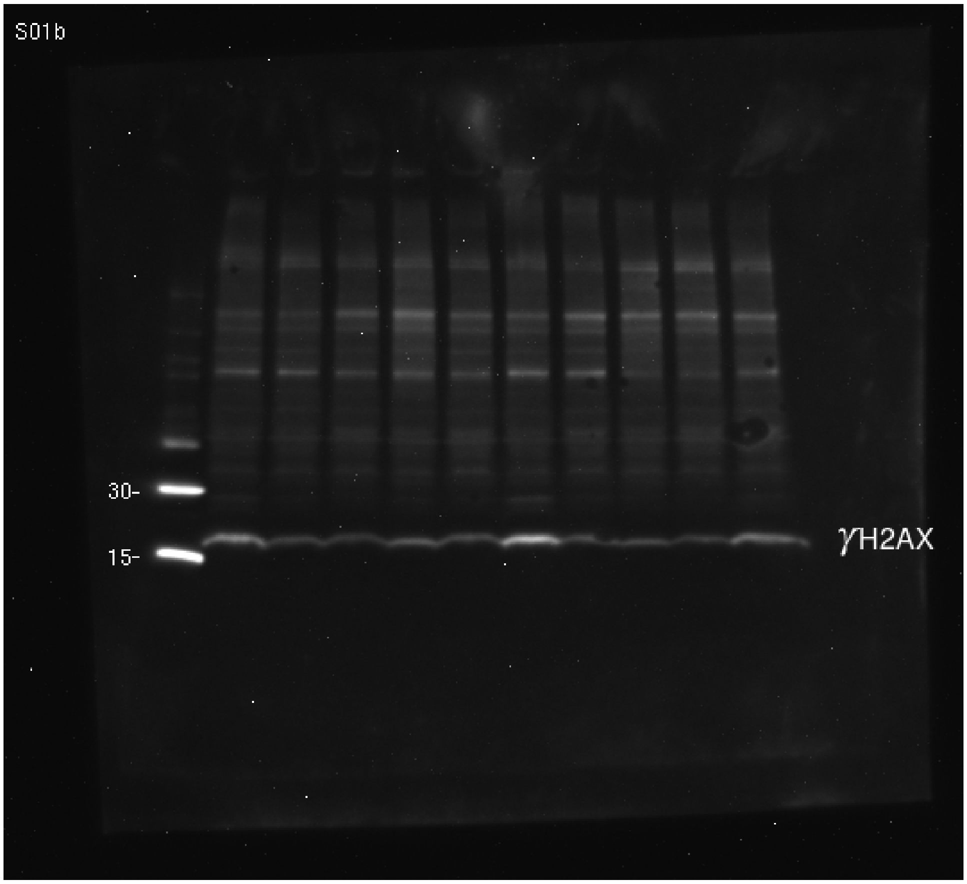
S01b
30-
15-
γH2AX

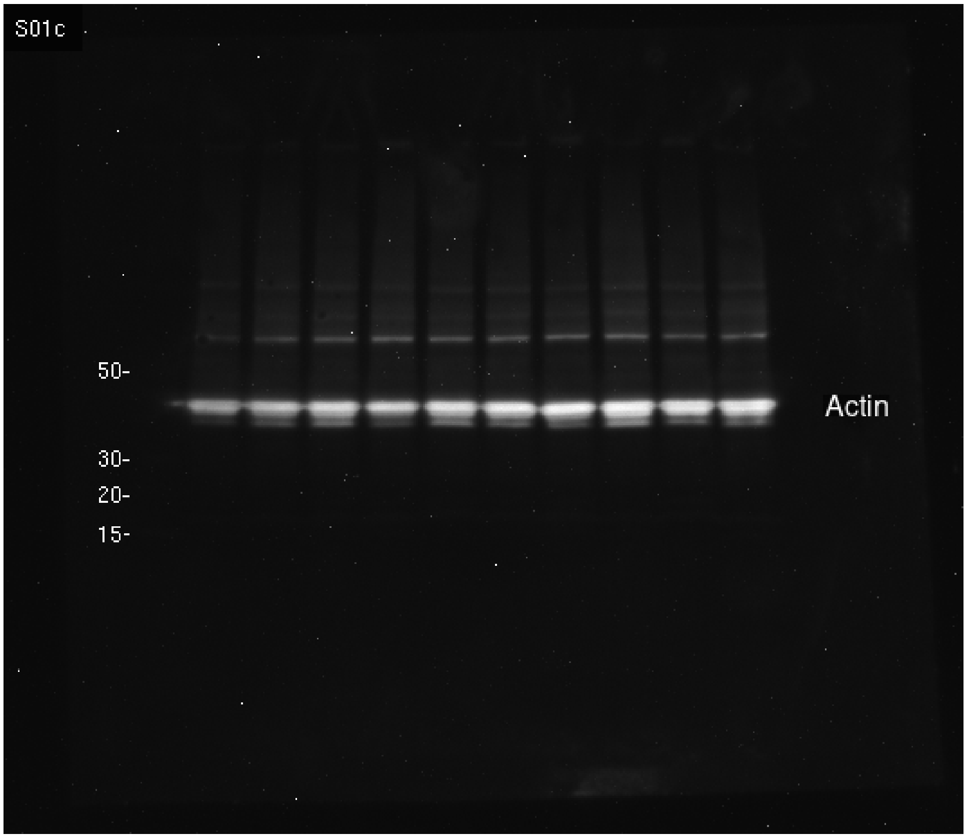
S01c
50-
Actin
30-
20-
15-

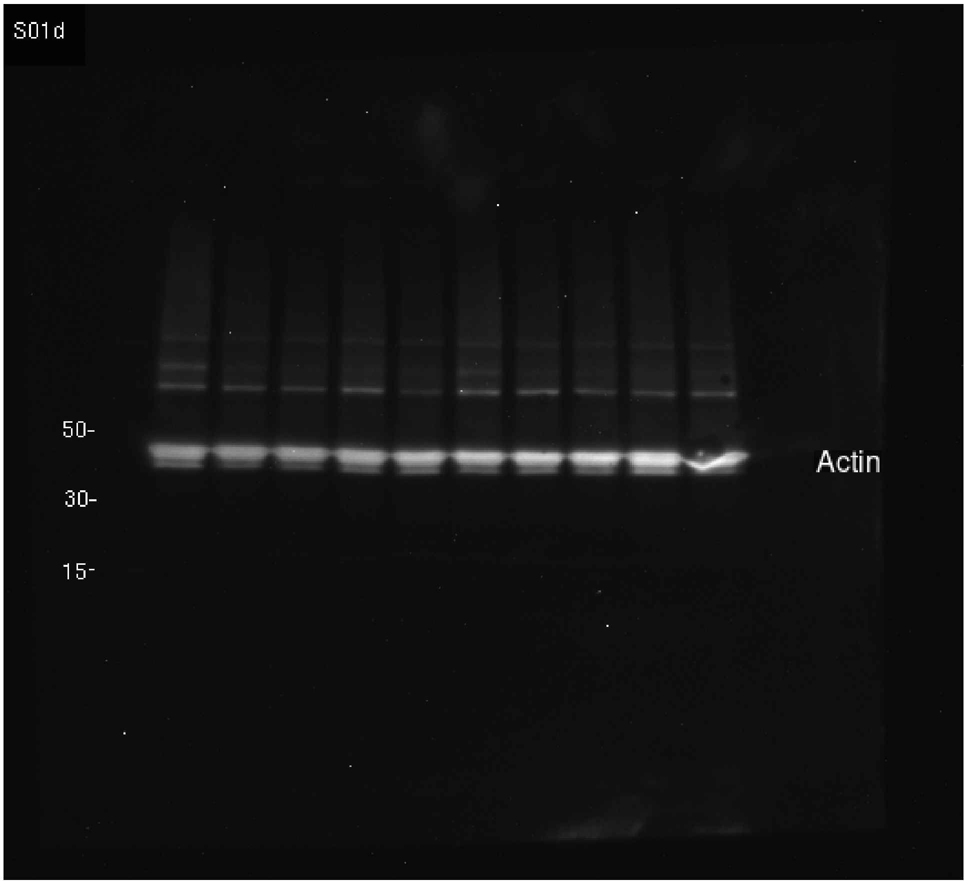
S01d
50-
Actin
30-
15-

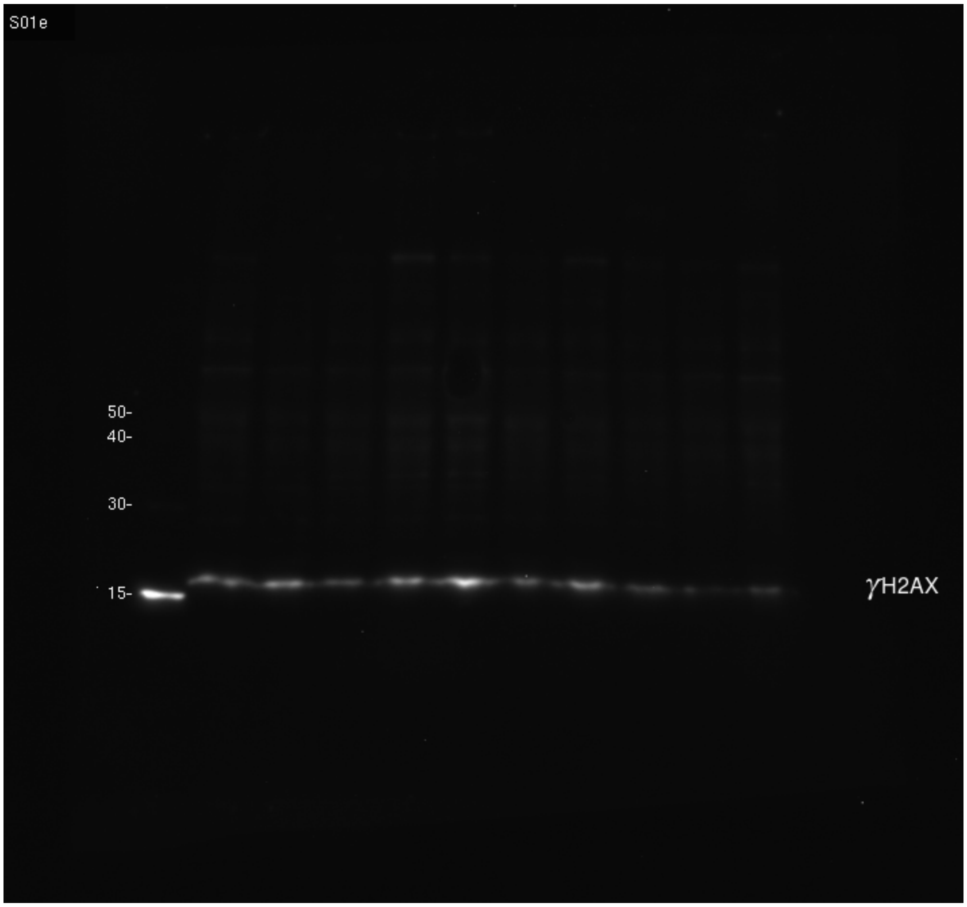
S01e
50-
40-
30-
15-
γH2AX

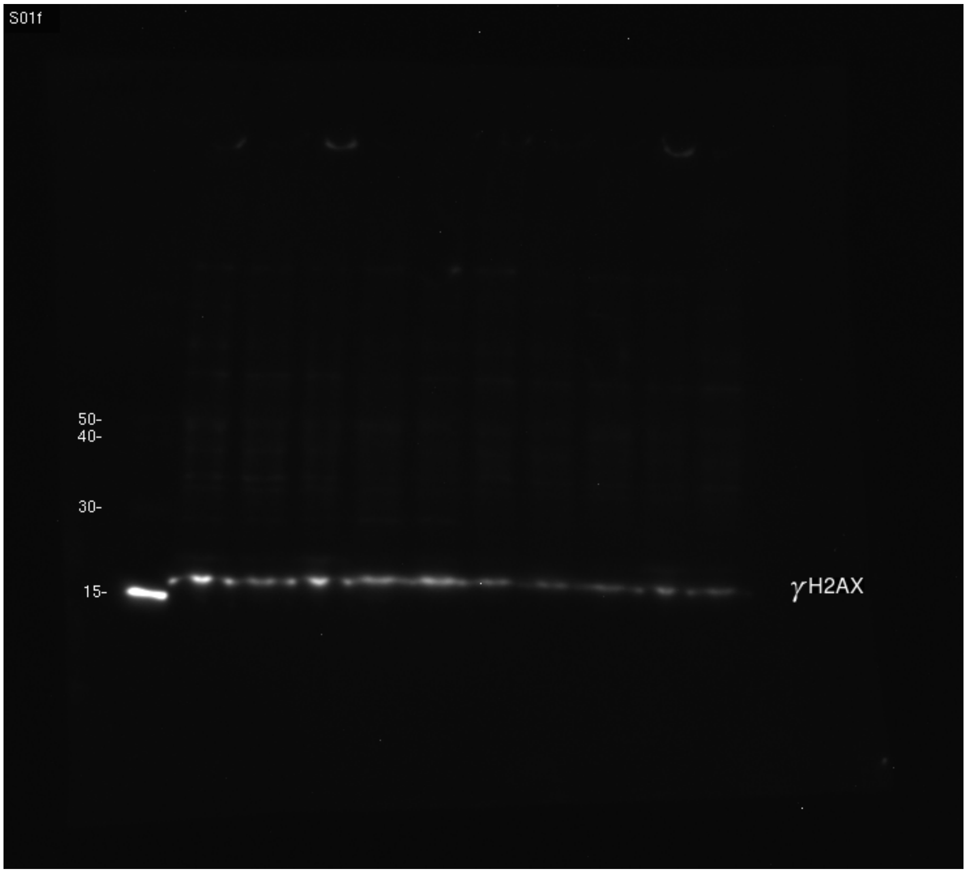
S01f
50-
40-
30-
15-
γH2AX

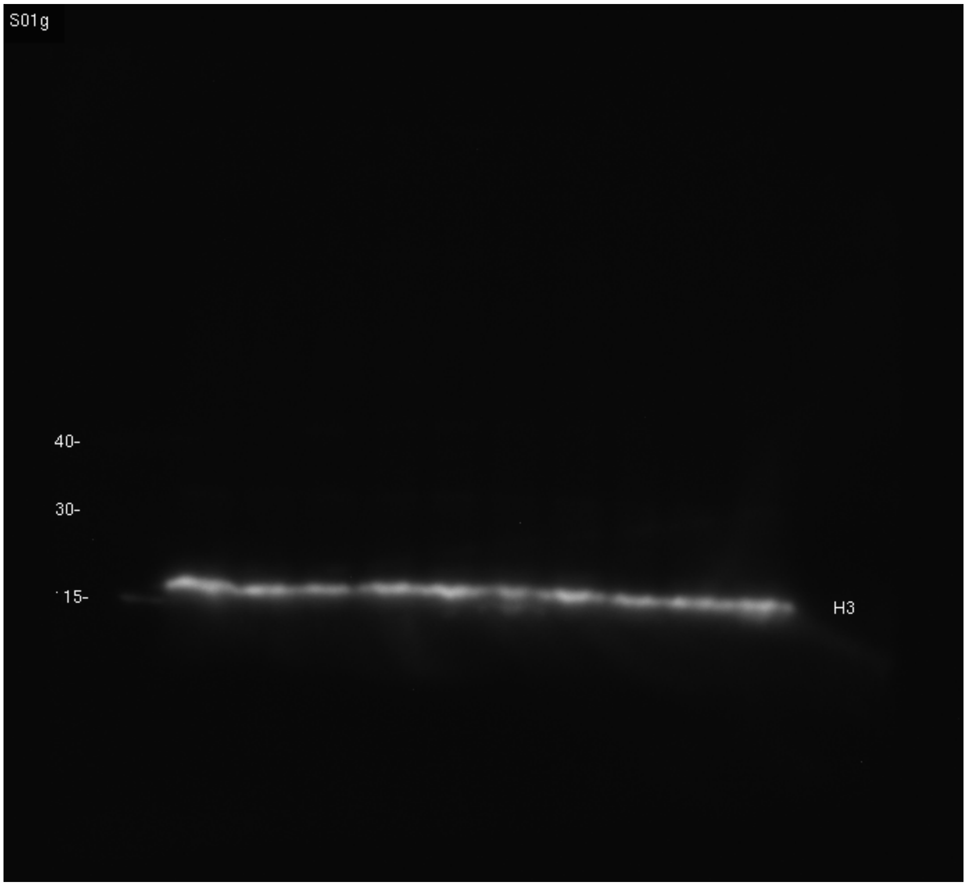
S01g
40-
30-
15-
H3

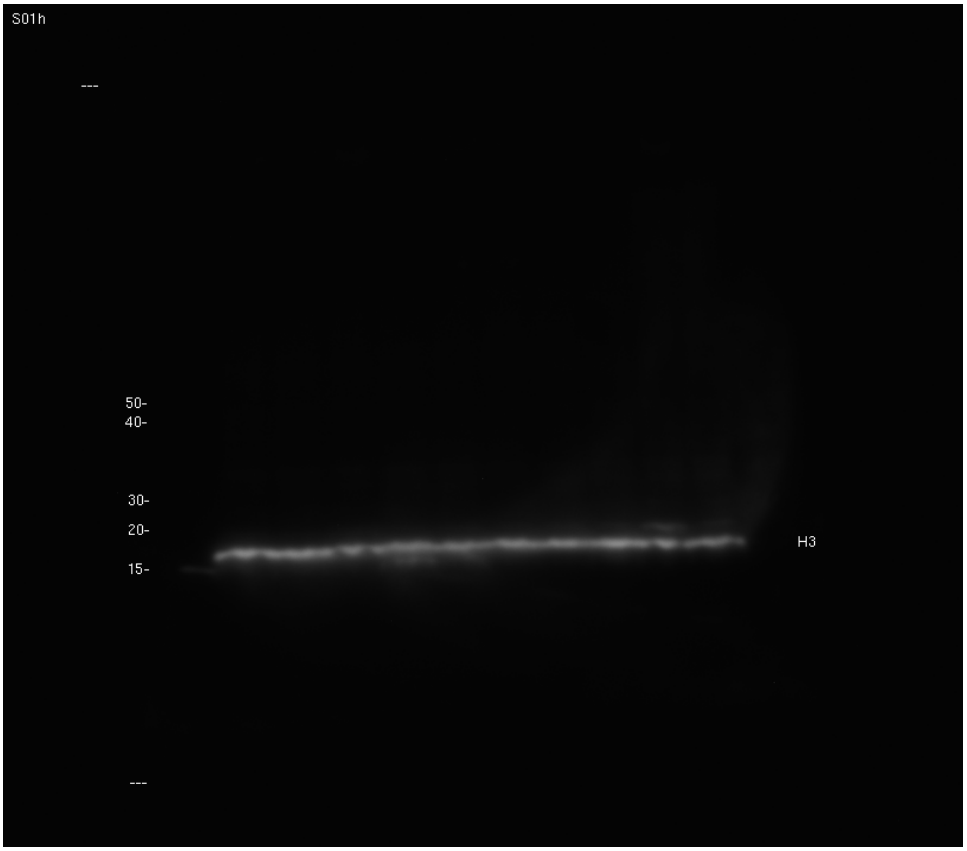
S01h

50-
40-
30-
20-
15-
H3

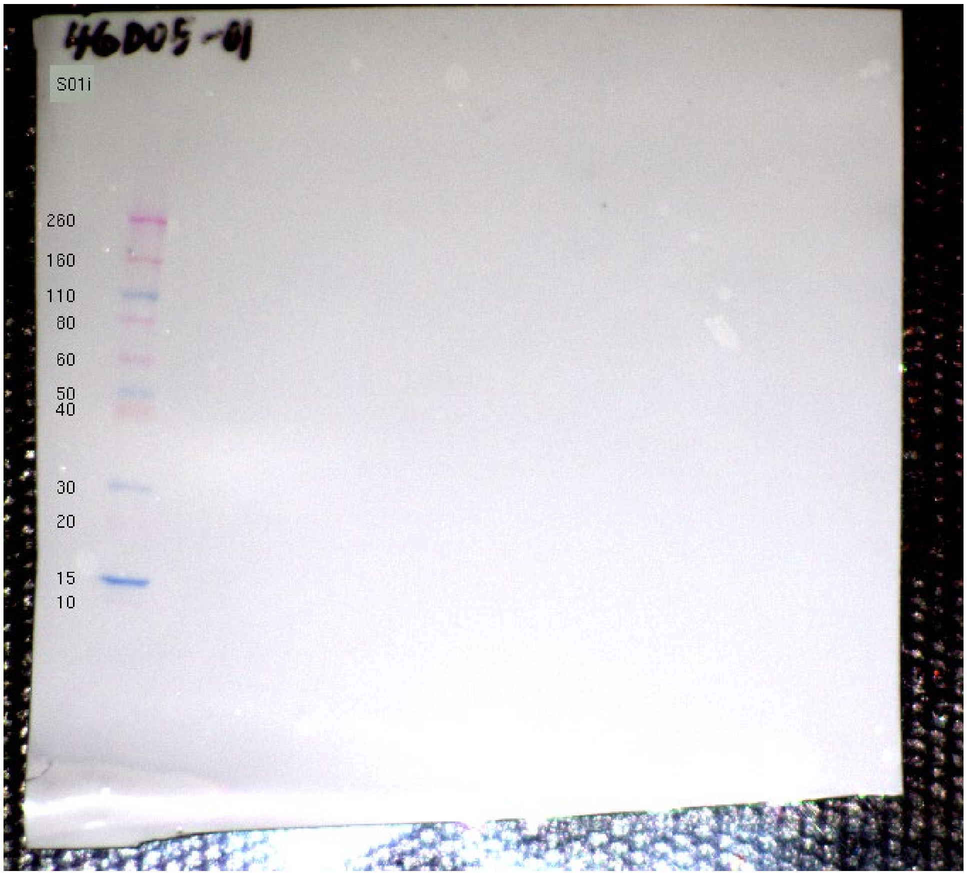
46D05-01
S01i
260
160
110
80
60
50
40
30
20
15
10

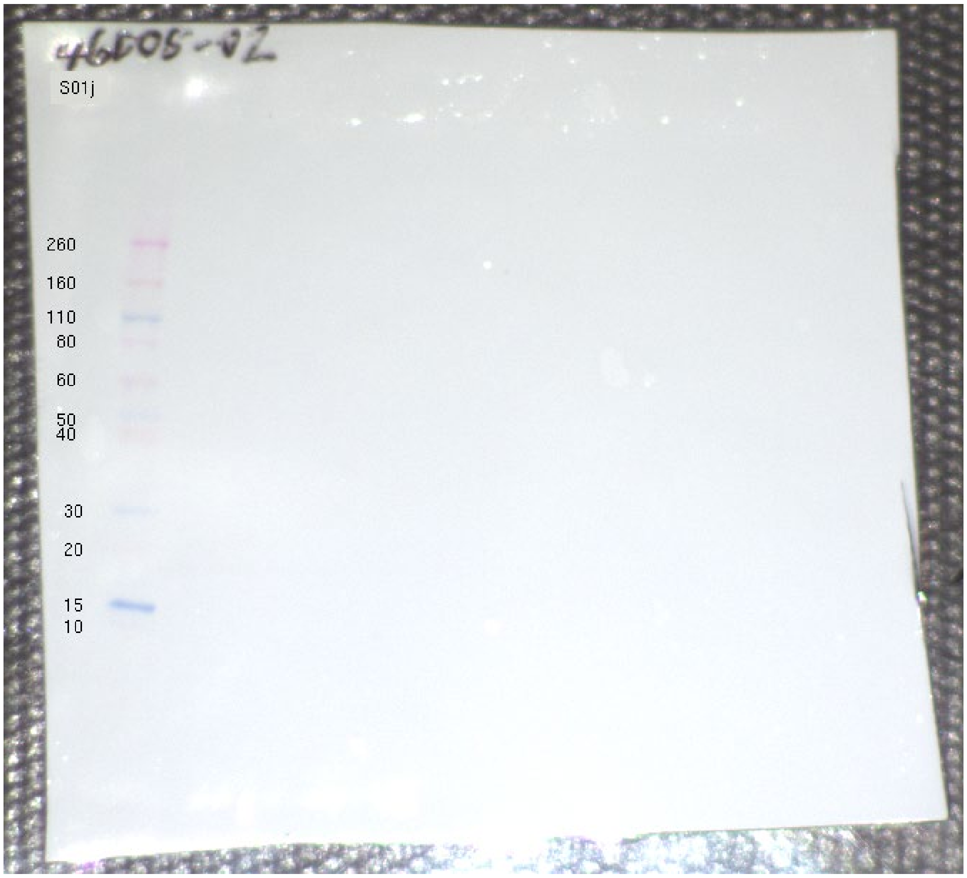
46D05-02
S01j
260
160
110
80
60
50
40
30
20
15
10

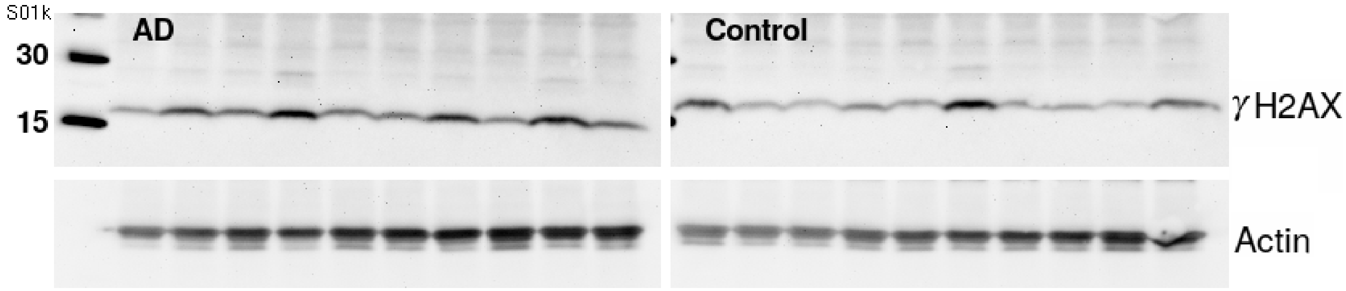
S01k
AD
Control
30
15
γH2AX
Actin

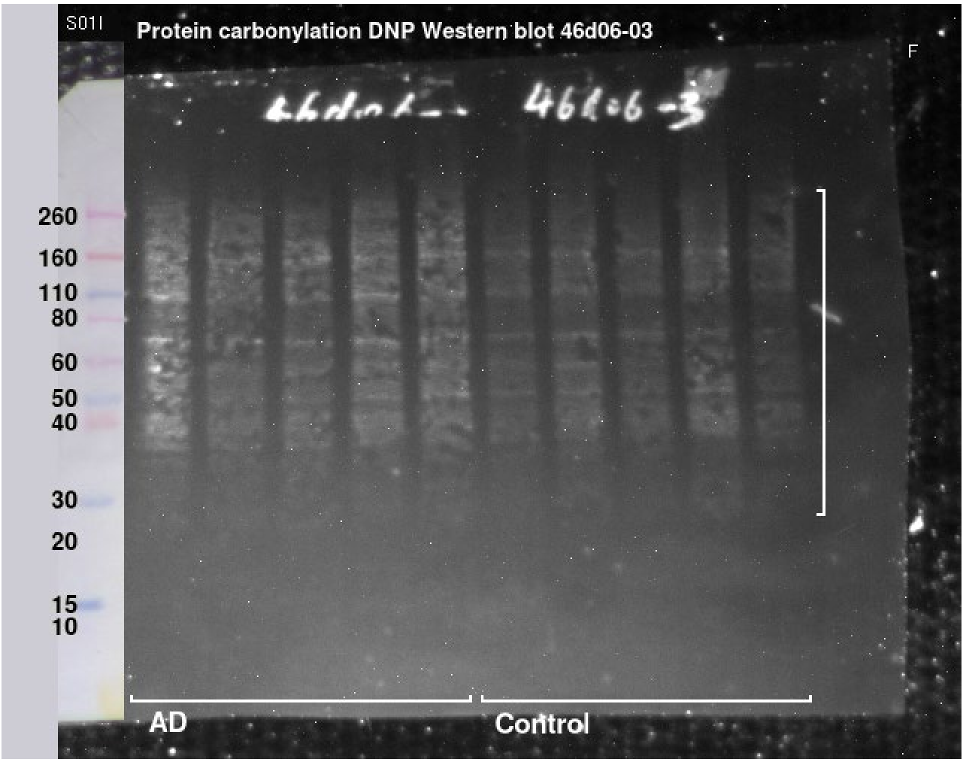
S01l
Protein carbonylation DNP Western blot 46d06-03
F
260
160
110
80
60
50
40
30
20
15
10
AD
Control

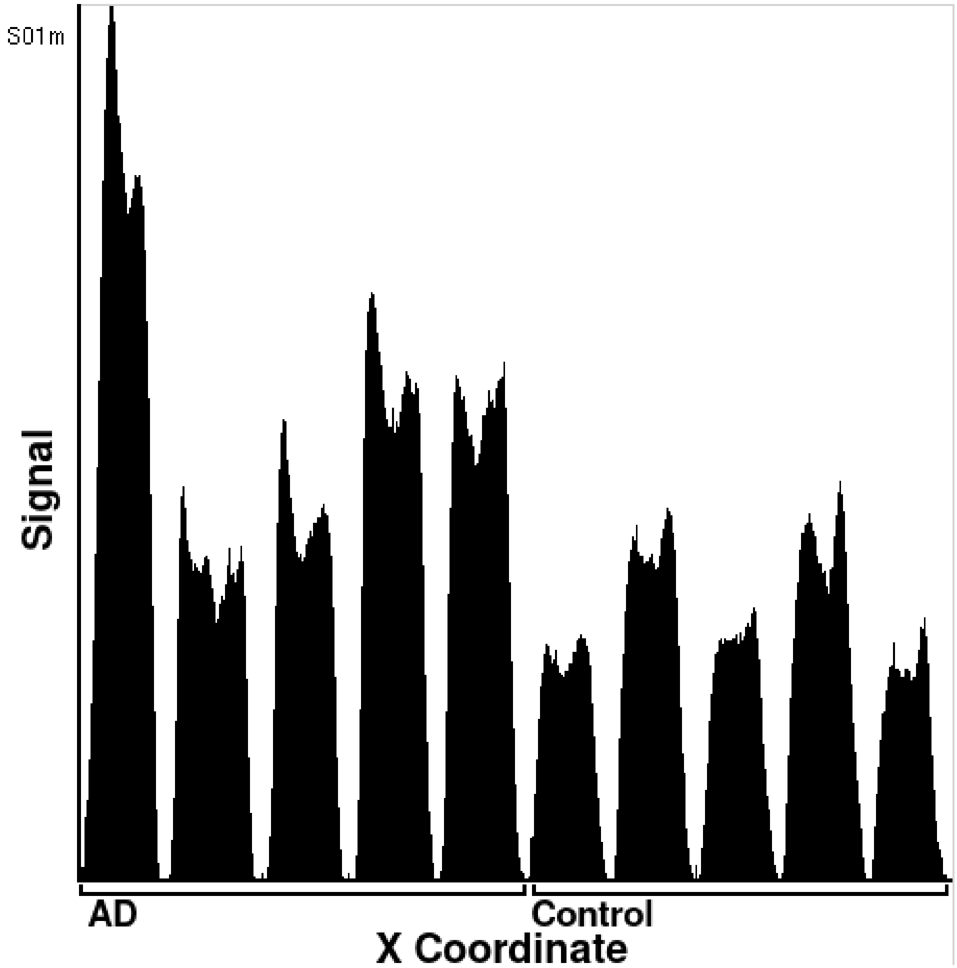
S01m
Signal
AD
Control
X Coordinate

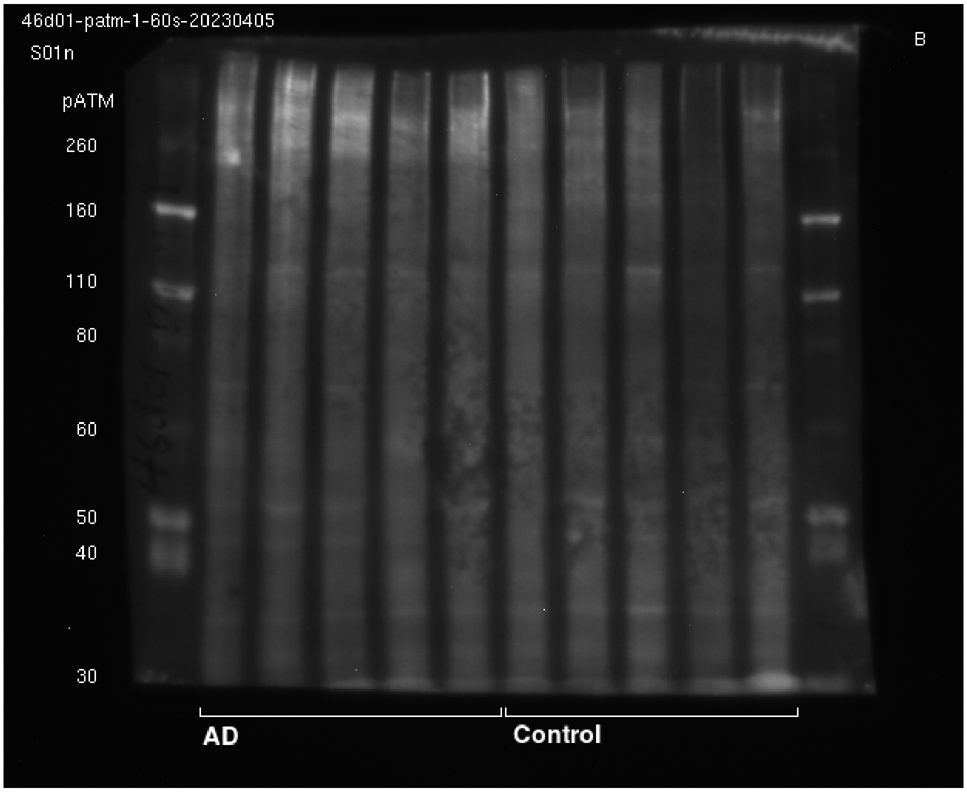
46d01-patm-1-60s-20230405
S01n
B
pATM
260
160
110
80
60
50
40
30
AD
Control

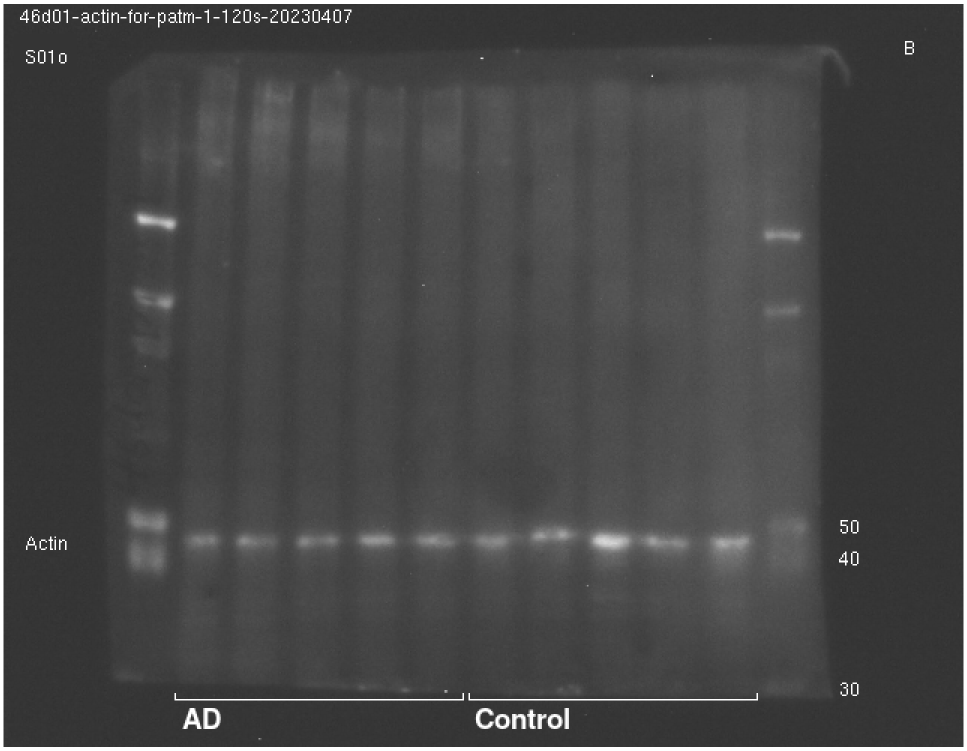
46d01-actin-for-patm-1-120s-20230407
S01o
B
Actin
50
40
30
AD
Control

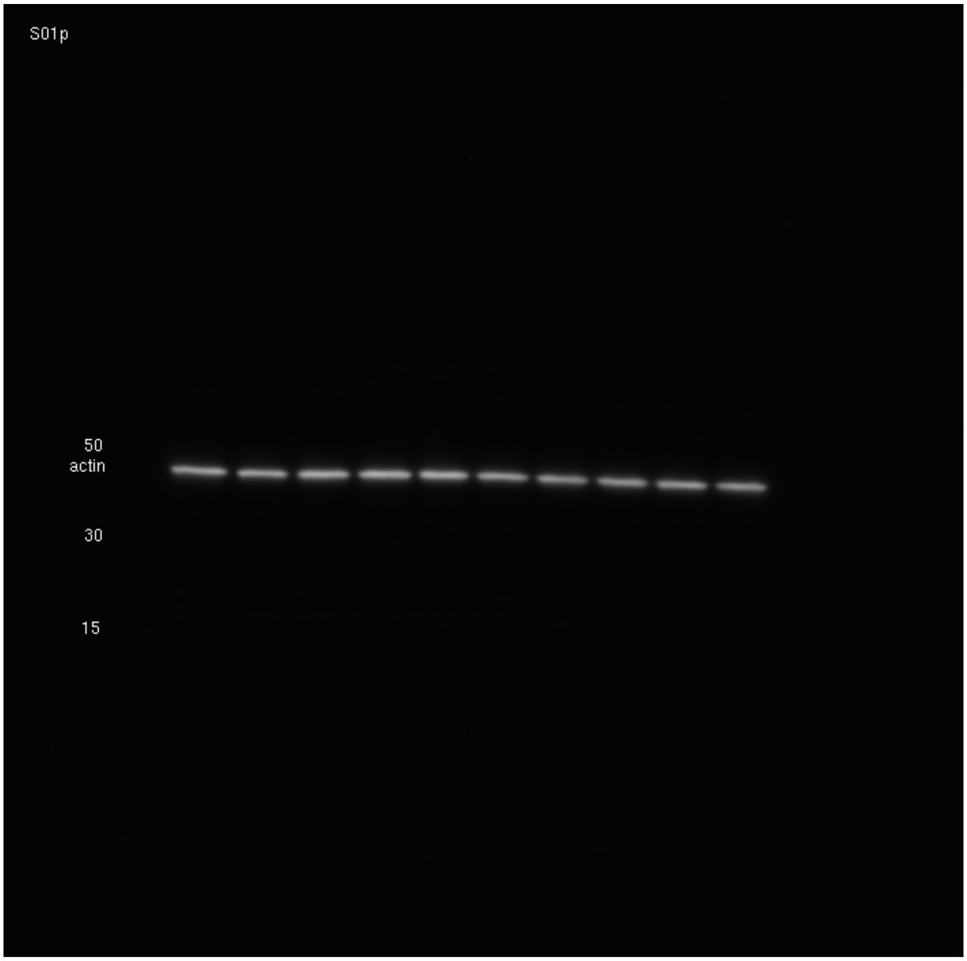
S01p
50
actin
30
15

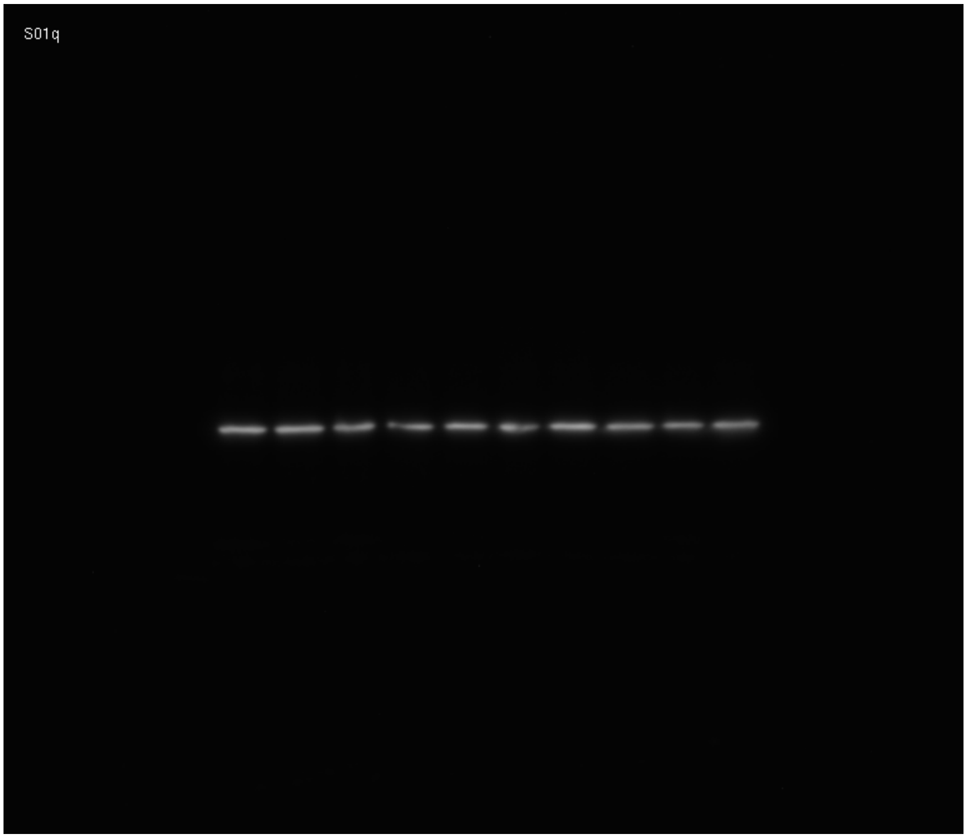
S01q

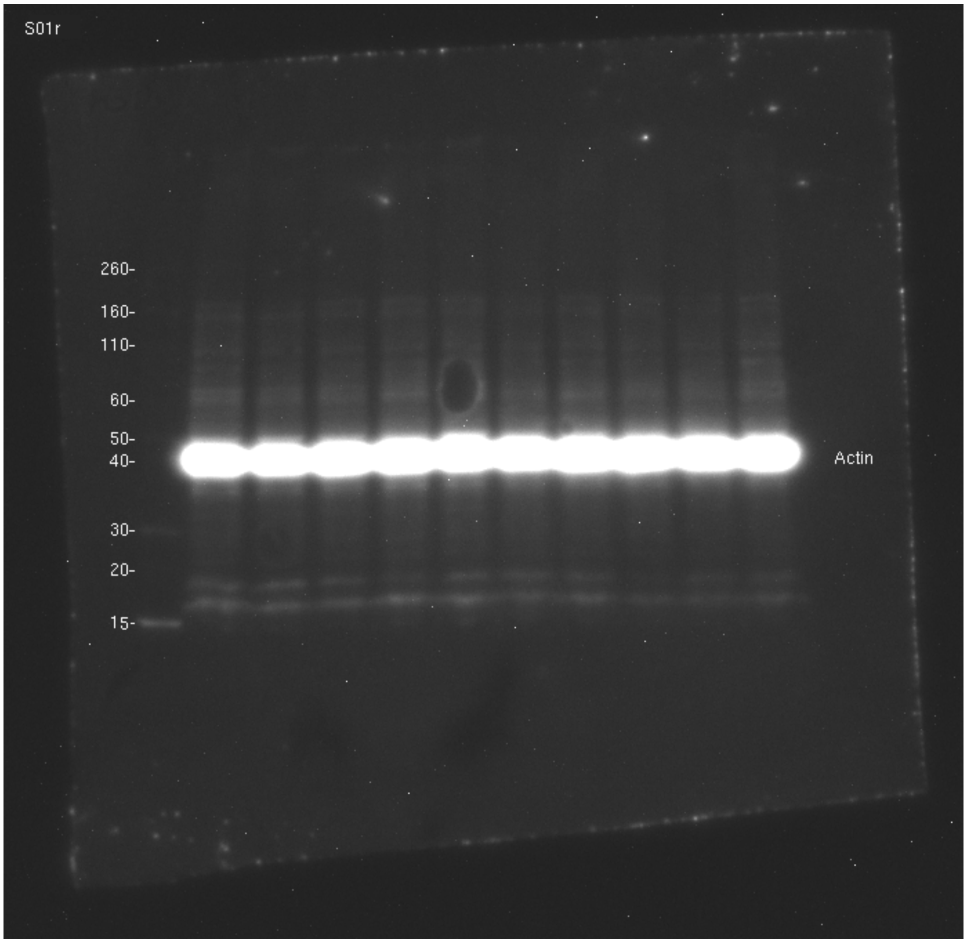
S01r
260-
160-
110-
60-
50-
40-
30-
20-
15-
Actin

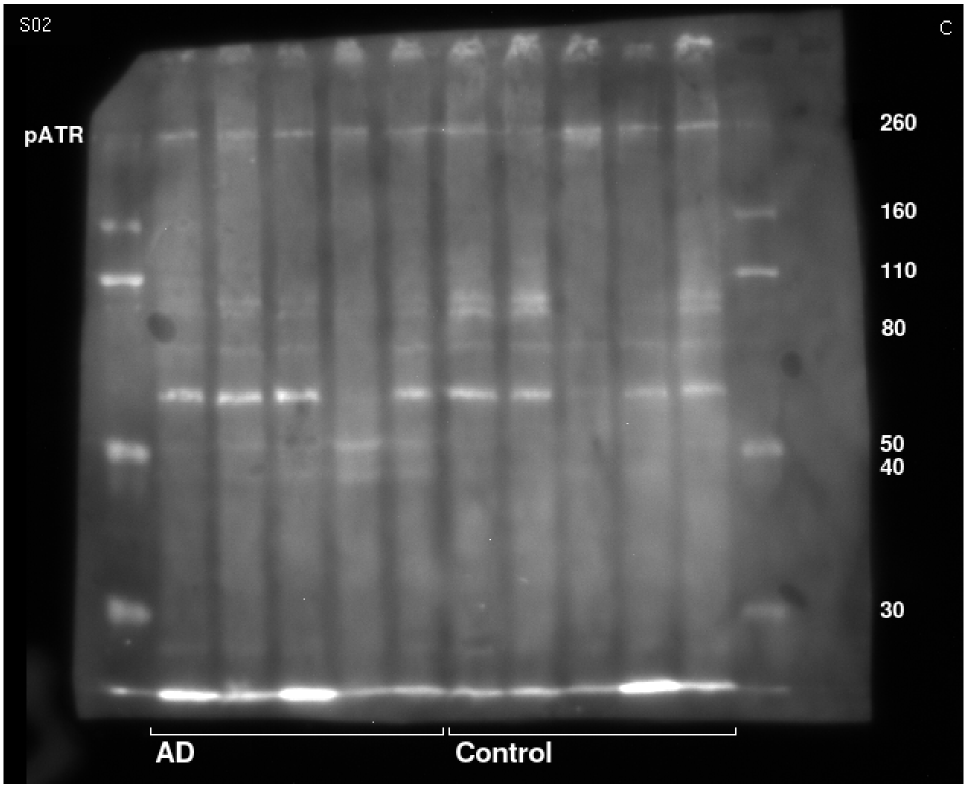

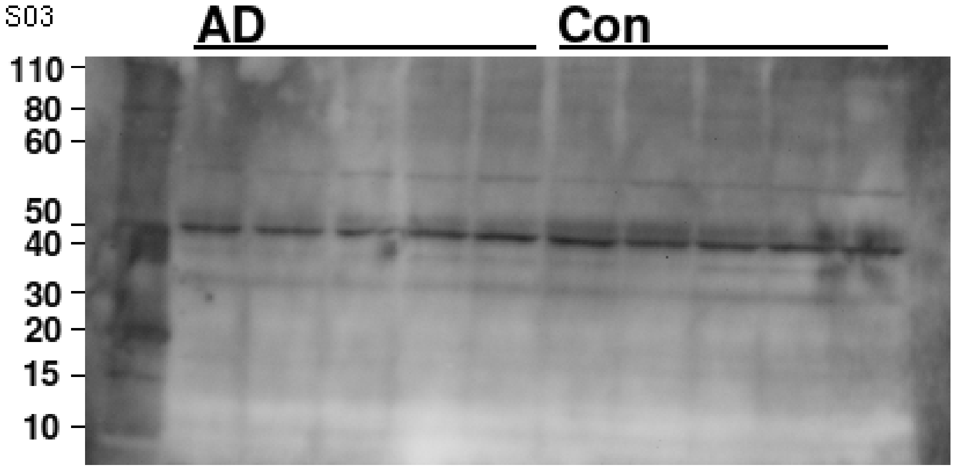

45i01-01-nuclei-p53ps15-60s

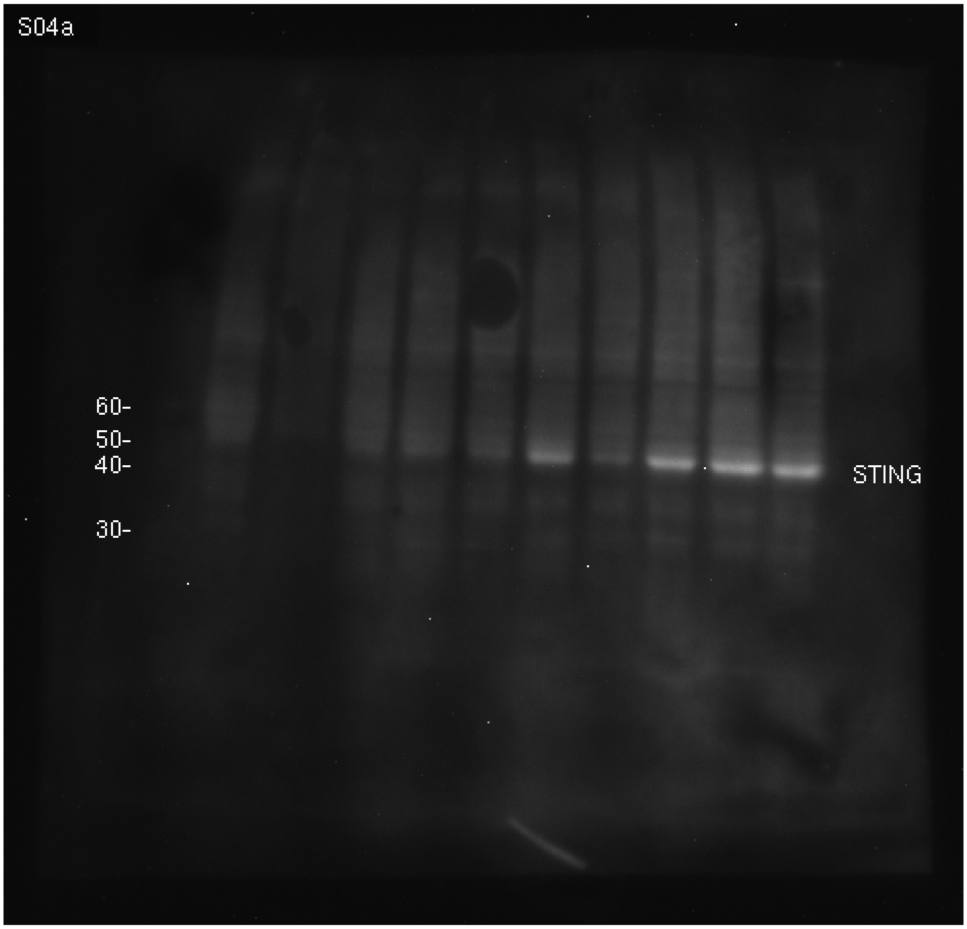
S04a
60-
50-
40-
30-
STING

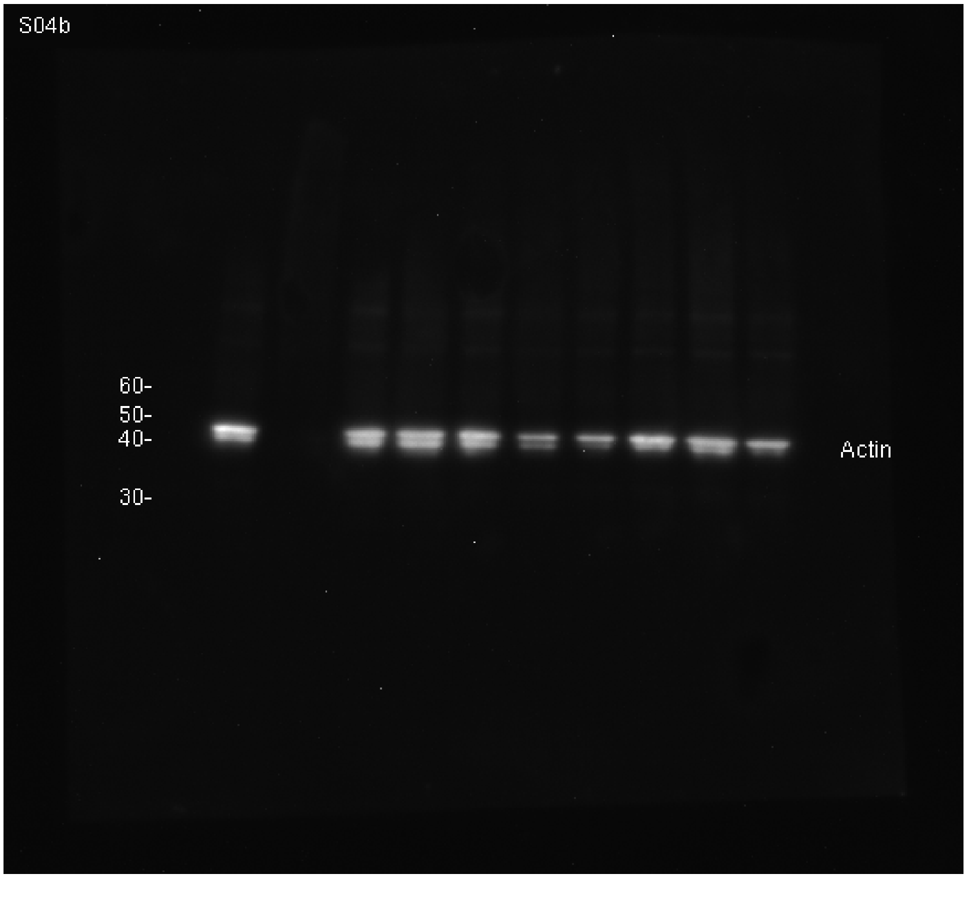
S04b
60-
50-
40-
30-
Actin

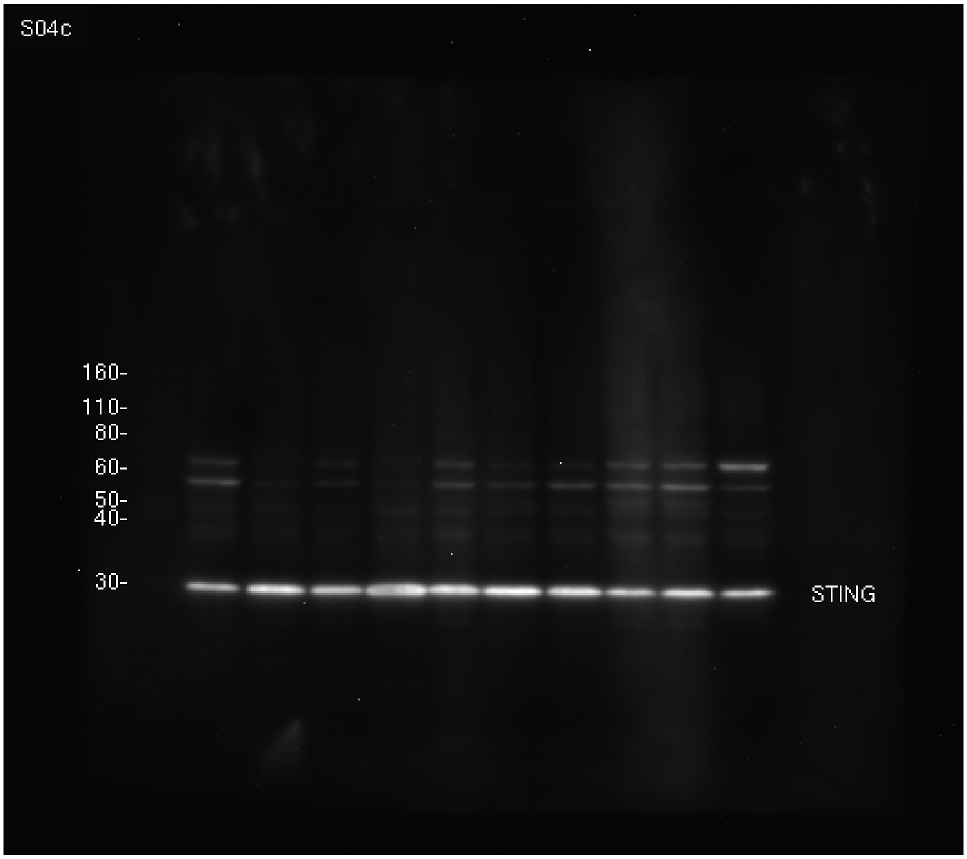
S04c
160-
110-
80-
60-
50-
40-
30-
STING

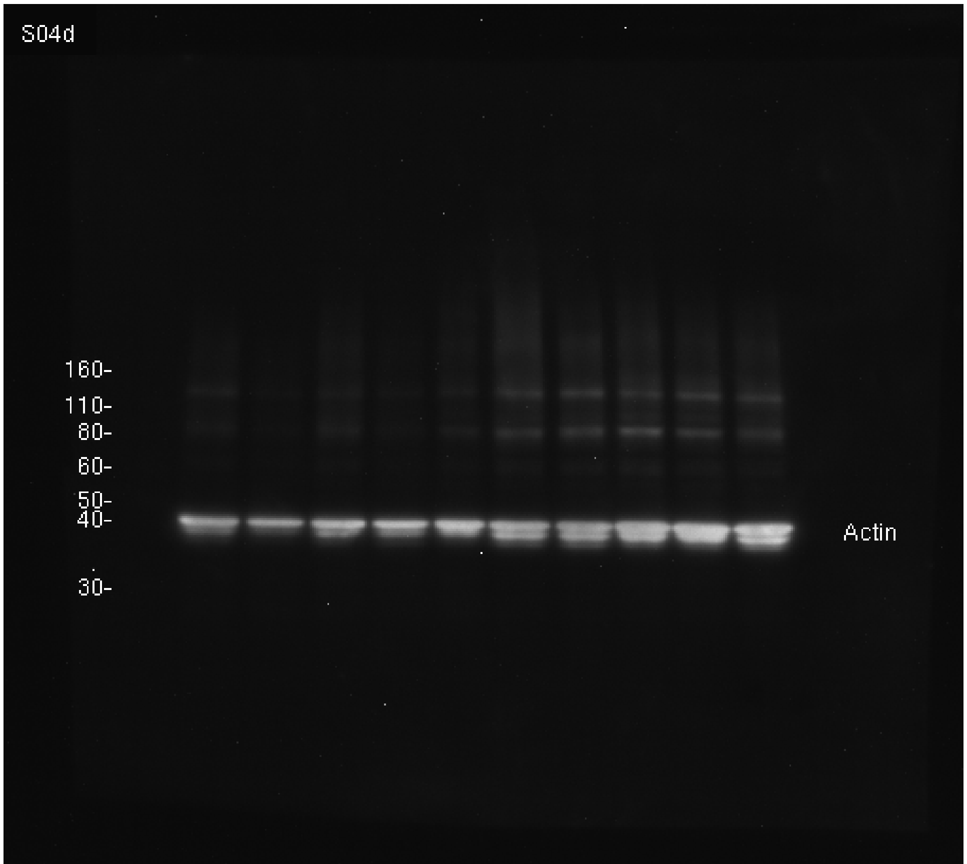
S04d
160-
110-
80-
60-
50-
40-
30-
Actin

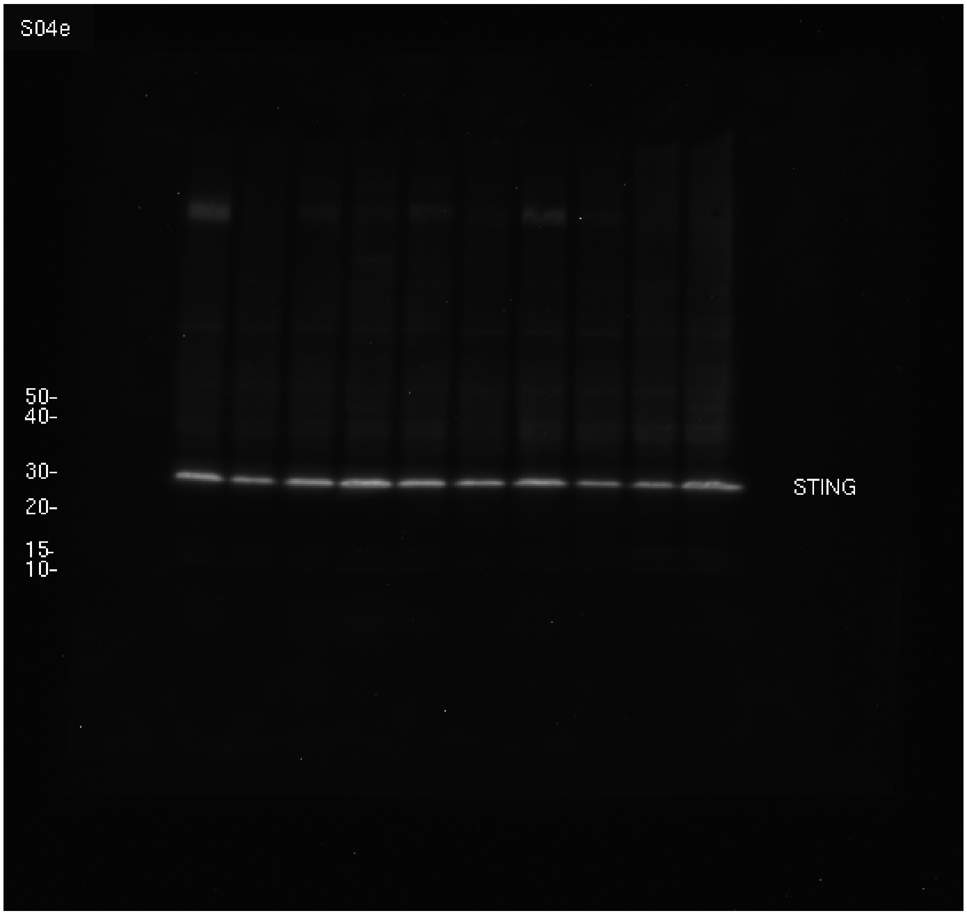
S04e
50-
40-
30-
20-
15-
10-
STING

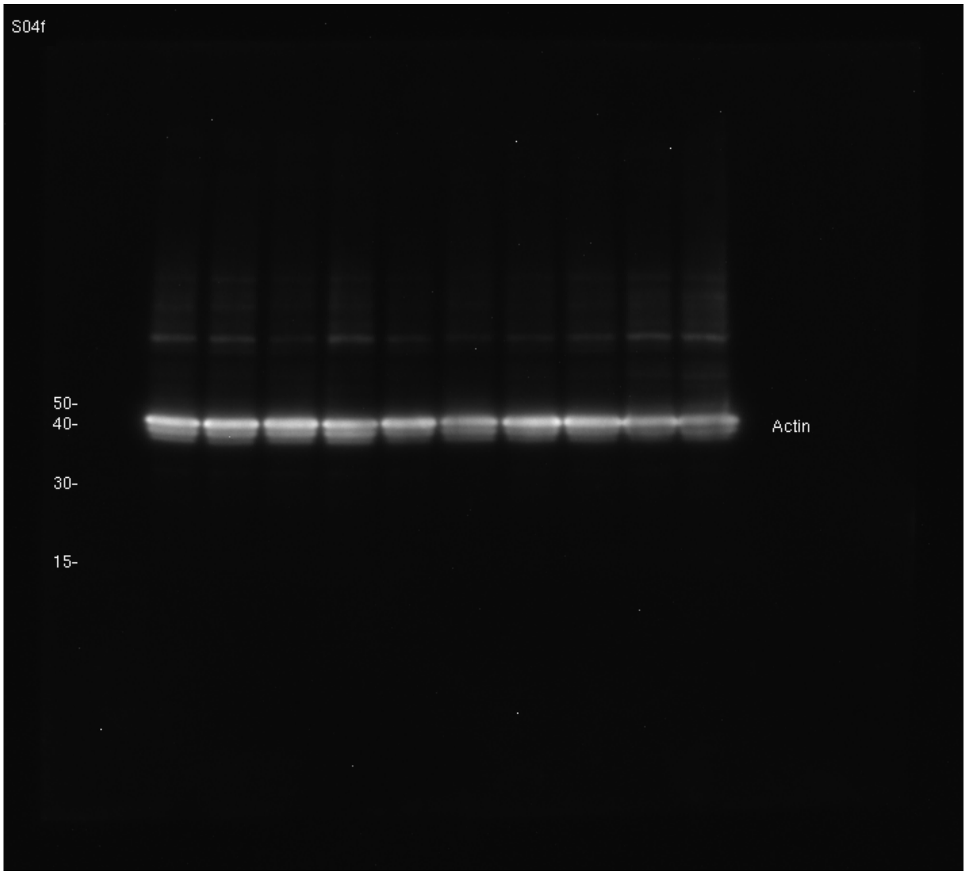
S04f
50-
40-
30-
15-
Actin

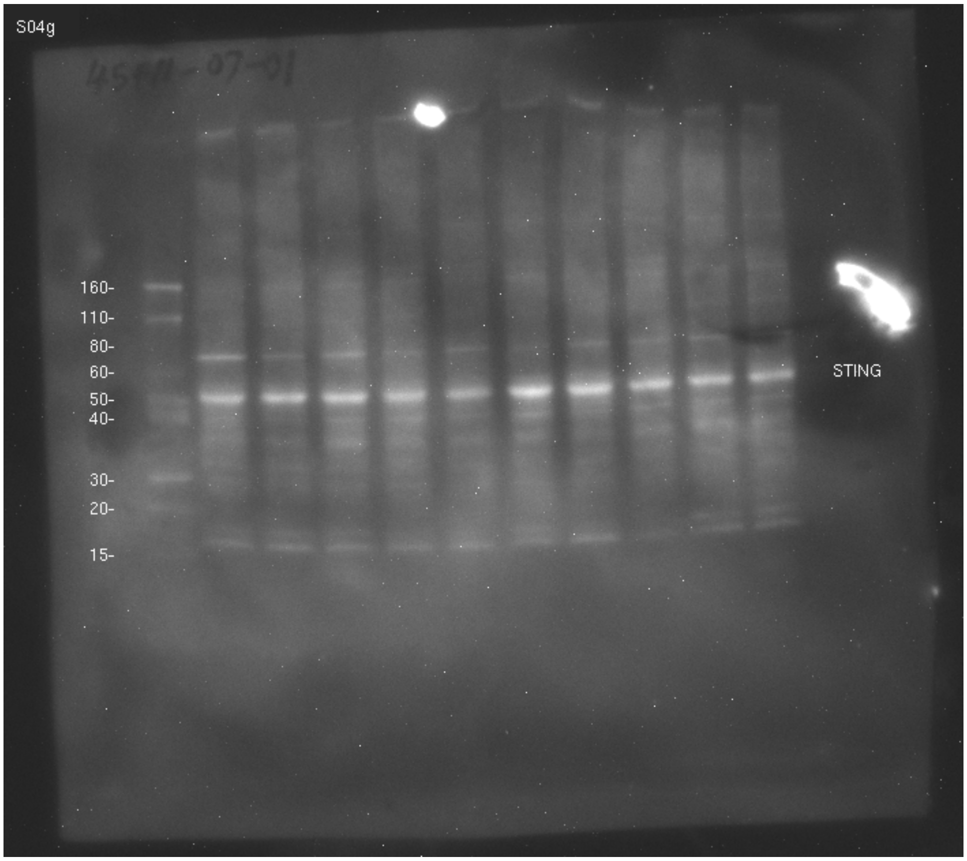
S04g
160-
110-
80-
60-
50-
40-
30-
20-
15-
STING

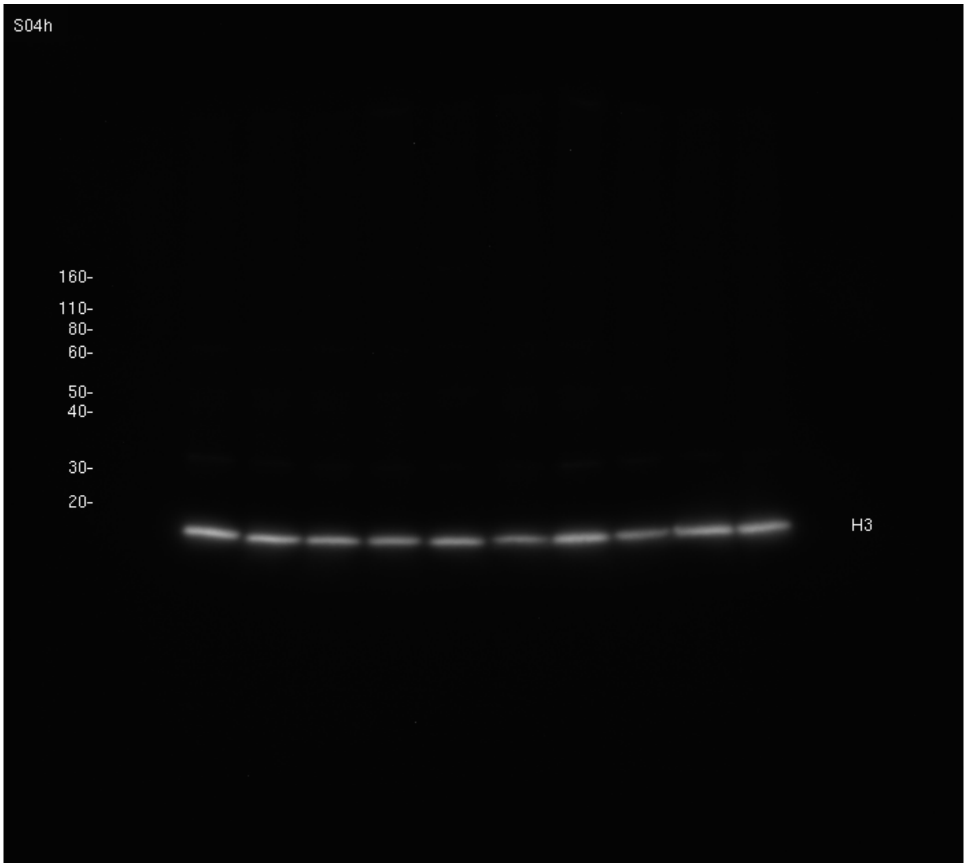
S04h
160-
110-
80-
60-
50-
40-
30-
20-
H3

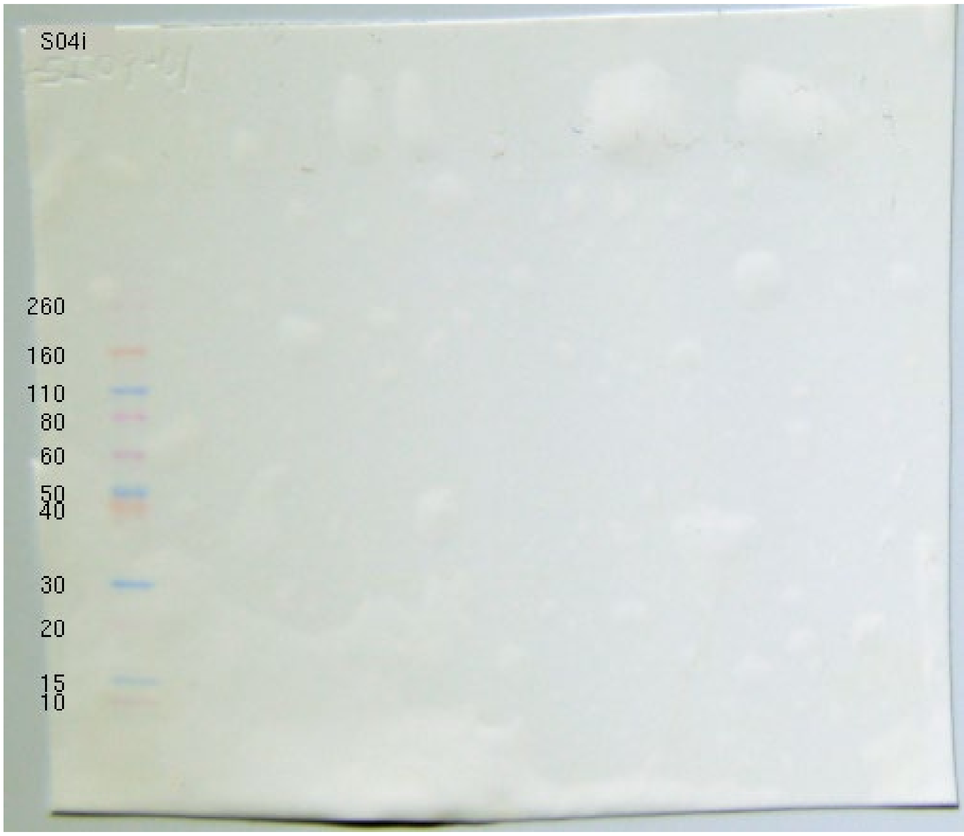

45i11-07-01 sting wb

S04j

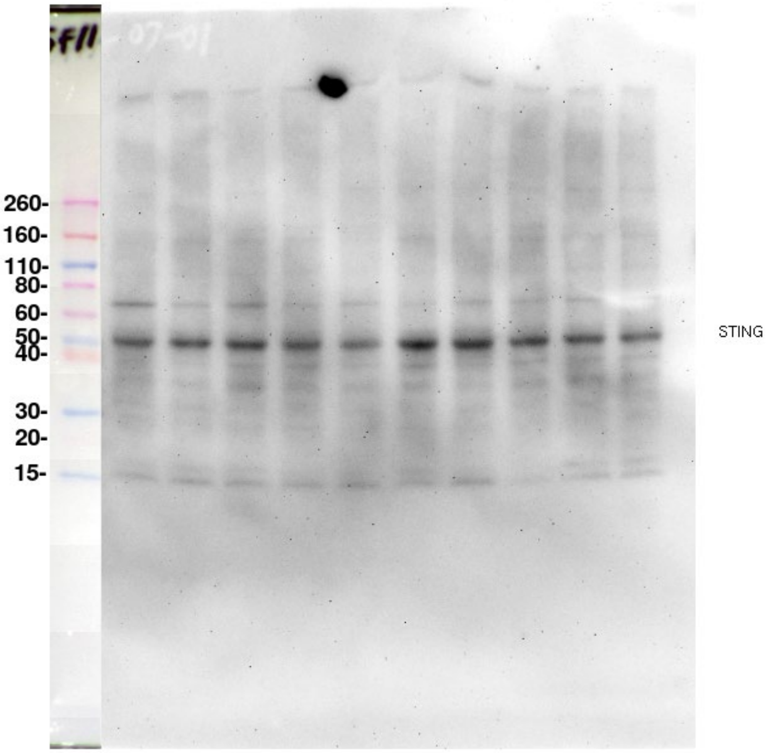

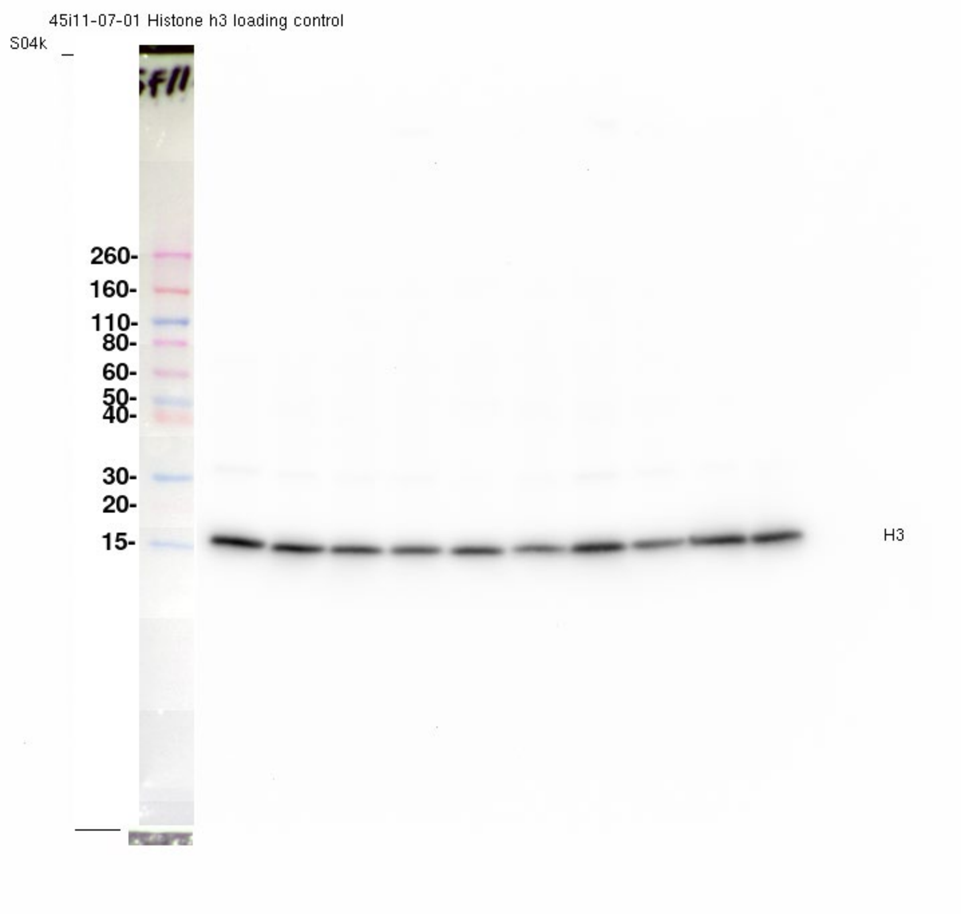
45i11-07-01 Histone h3 loading control
S04k
260-
160-
110-
80-
60-
50-
40-
30-
20-
15-
H3

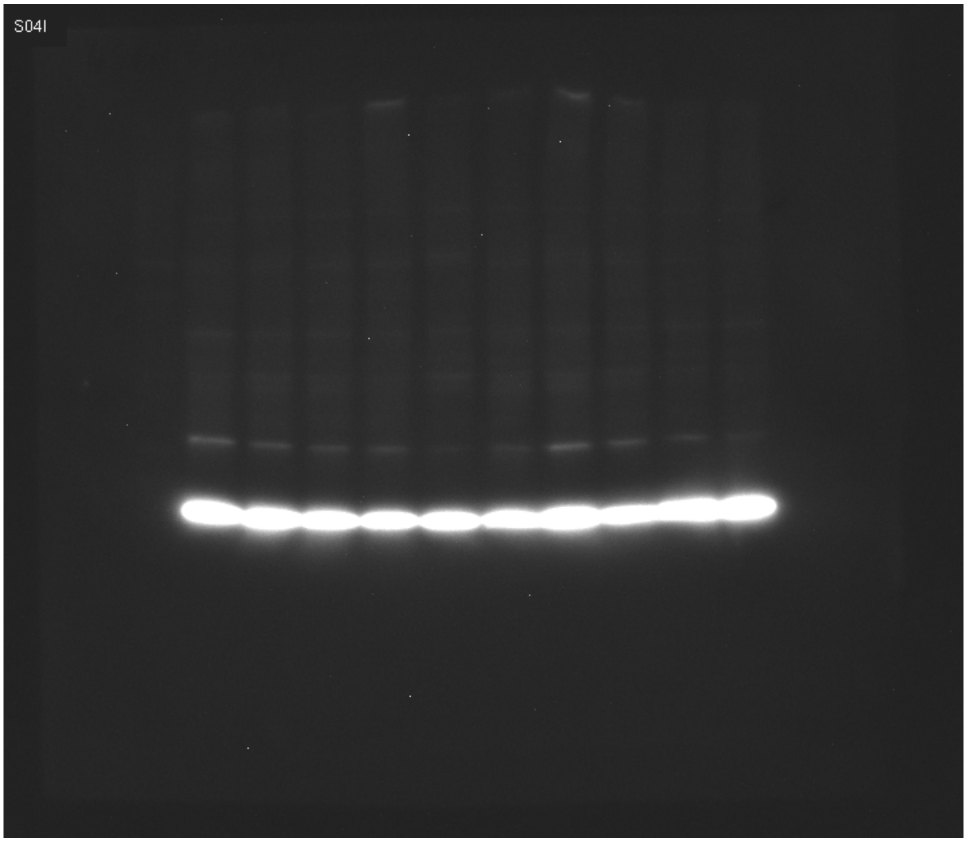
S04l

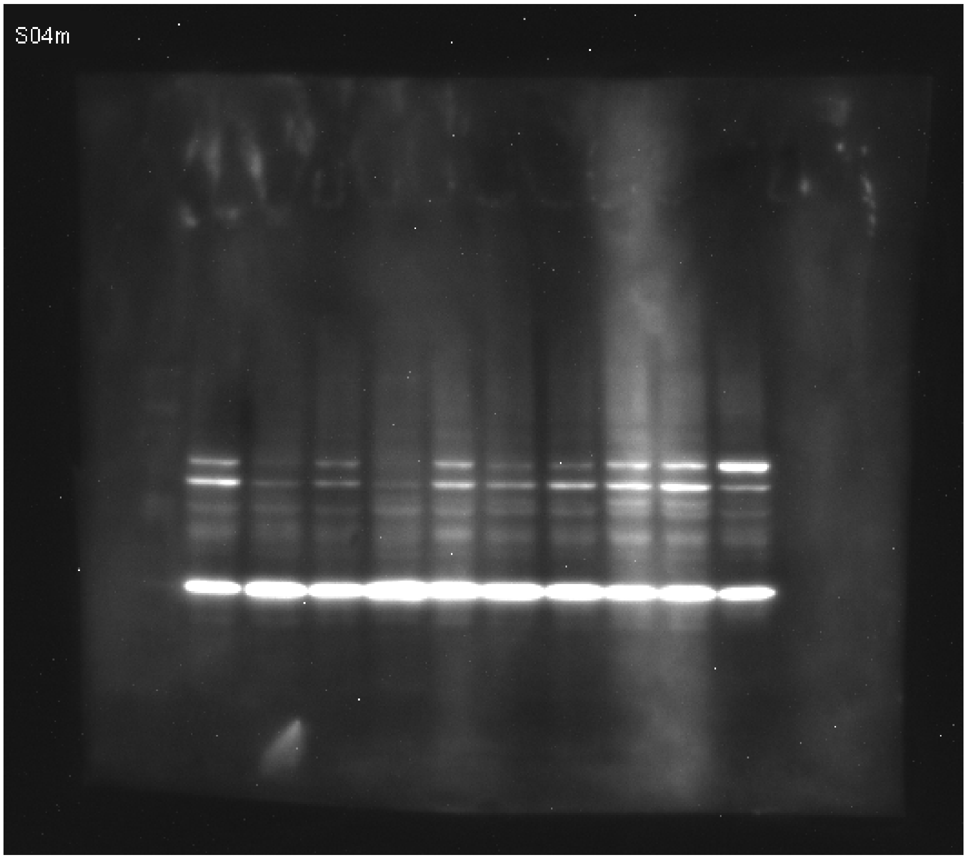
S04m

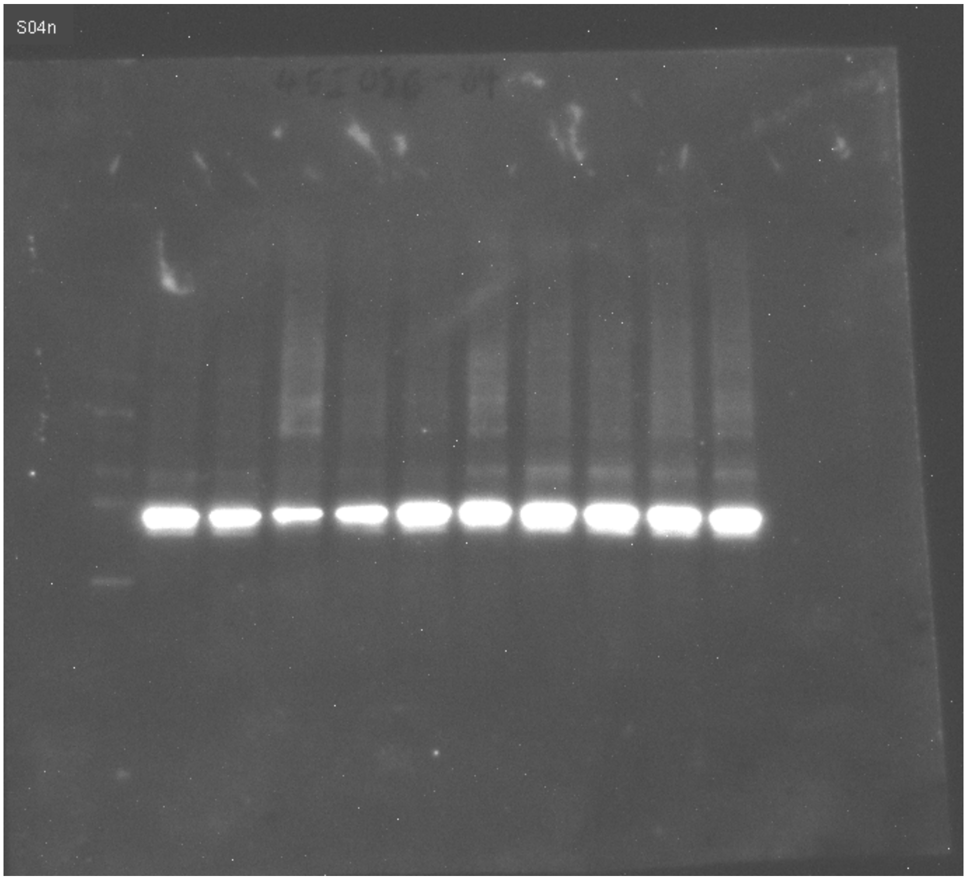
S04n

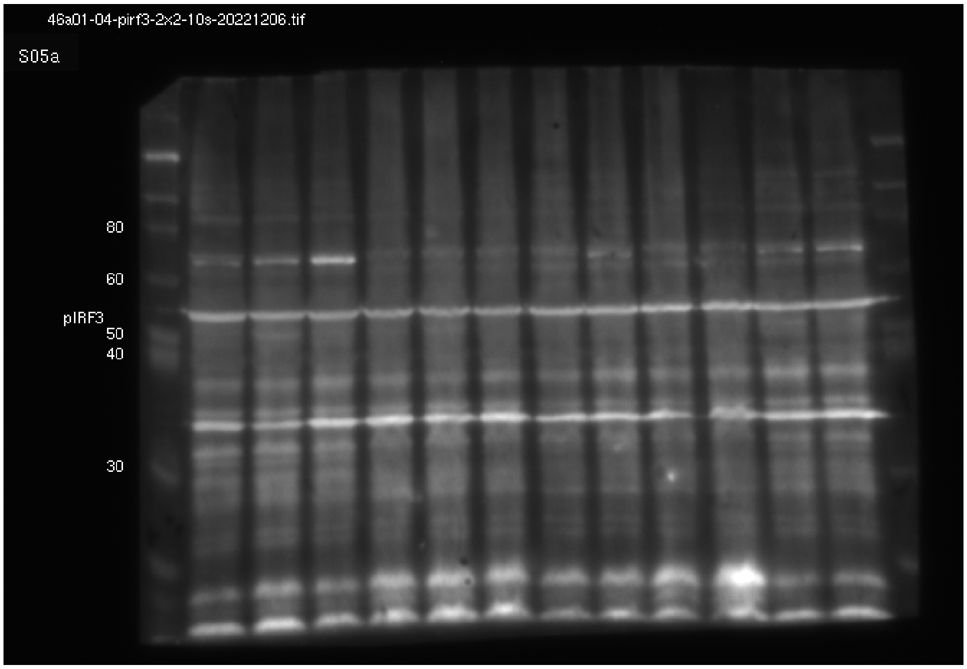
46a01-04-pirf3-2x2-10s-20221206.tif
S05a
80
60
pIRF3
50
40
30

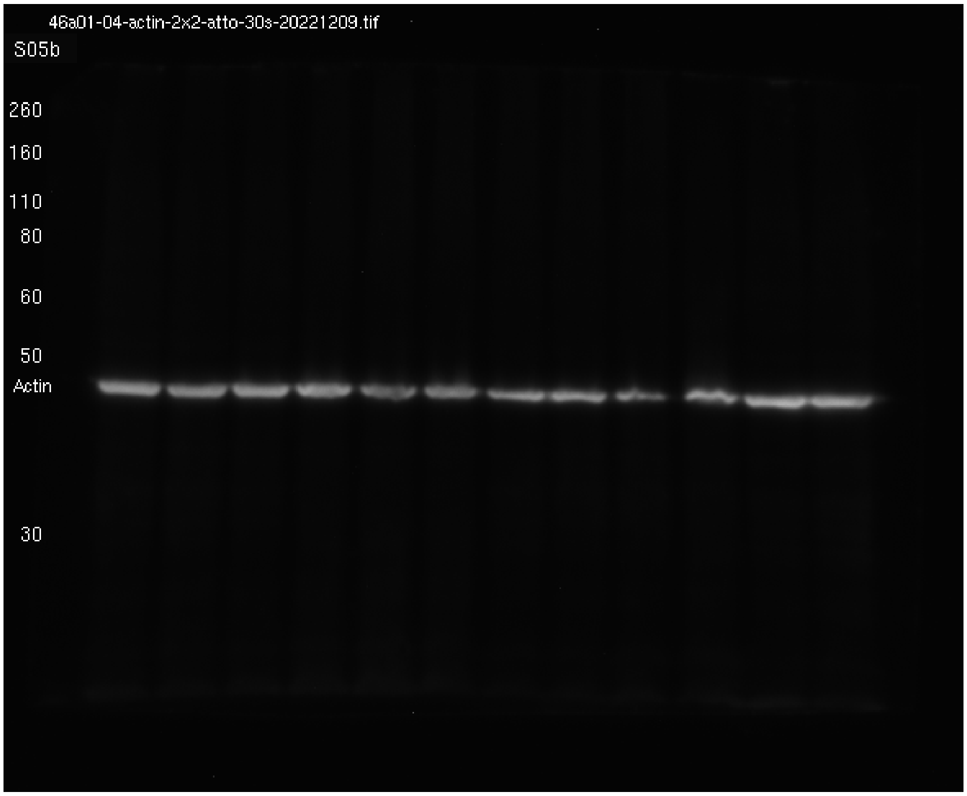
46a01-04-actin-2x2-atto-30s-20221209.tif
S05b
260
160
110
80
60
50
Actin
30

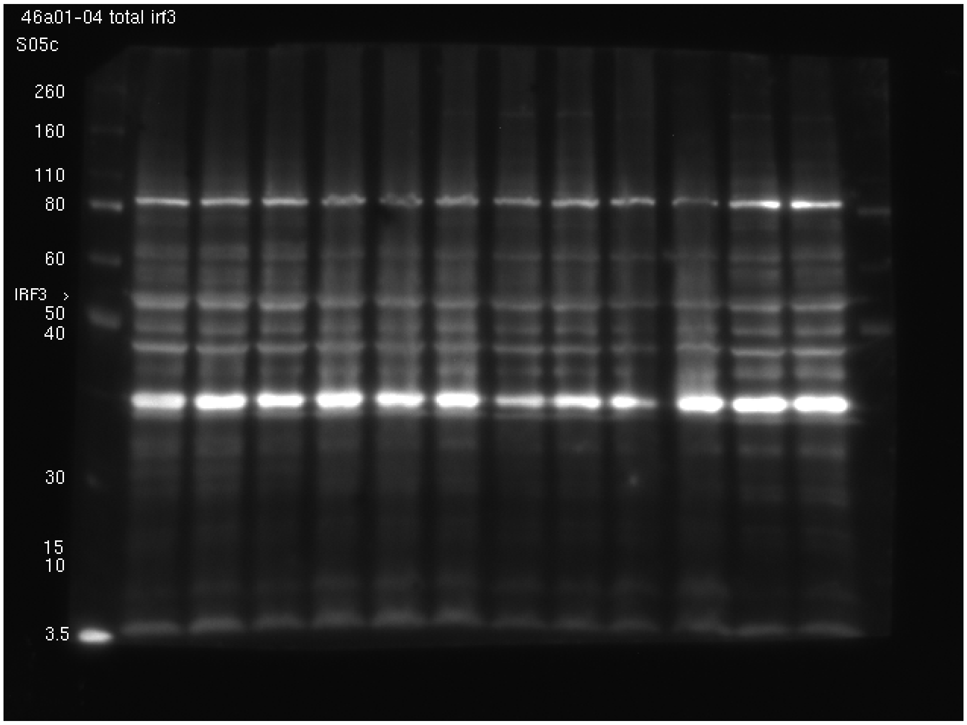
46a01-04 total irf3
S05c
260
160
110
80
60
IRF3 >
50
40
30
15
10
3.5

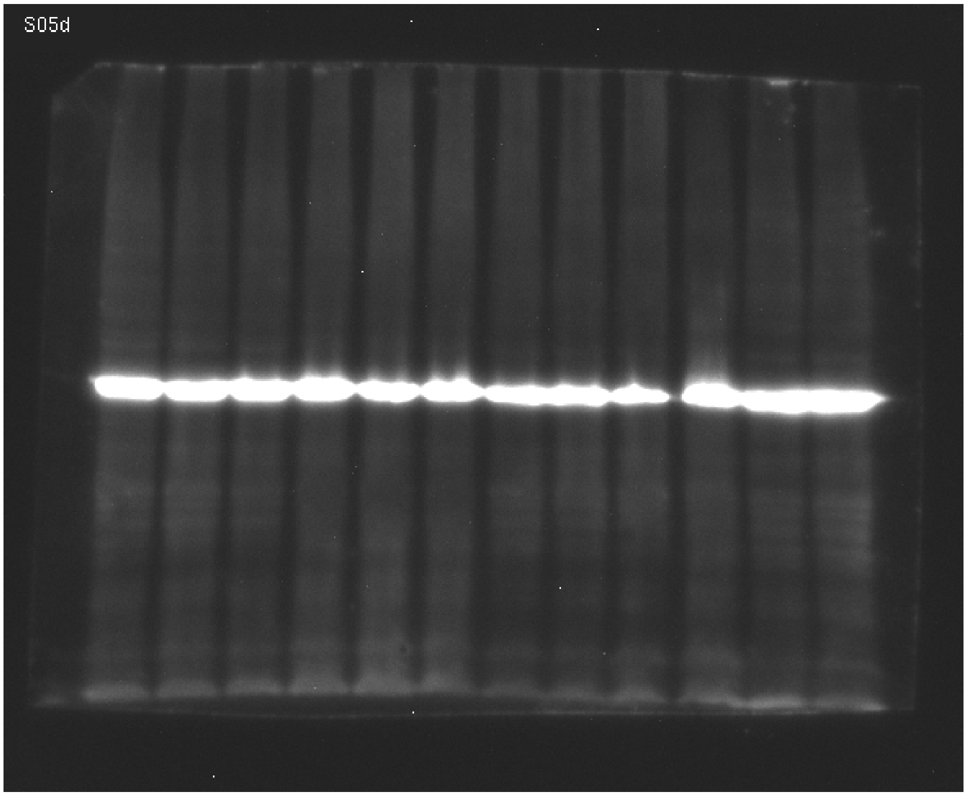
S05d
